# Feeder-free culture of human pluripotent stem cells drives MDM4-mediated gain of chromosome 1q

**DOI:** 10.1016/j.stemcr.2024.06.003

**Published:** 2024-07-03

**Authors:** Dylan Stavish, Christopher J. Price, Gabriele Gelezauskaite, Haneen Alsehli, Kimberly A. Leonhard, Seth M. Taapken, Erik M. McIntire, Owen Laing, Bethany M. James, Jack J. Riley, Johanna Zerbib, Duncan Baker, Amy L. Harding, Lydia H. Jestice, Thomas F. Eleveld, Ad J.M. Gillis, Sanne Hillenius, Leendert H.J. Looijenga, Paul J. Gokhale, Uri Ben-David, Tenneille E. Ludwig, Ivana Barbaric

**Affiliations:** 1 Centre for Stem Cell Biology, School of Biosciences, The University of Sheffield, Sheffield, UK; 2 Neuroscience Institute, The University of Sheffield, Sheffield, UK; 3 INSIGNEO Institute, The University of Sheffield, Sheffield, UK; 4 WiCell Research Institute, Madison, WI, USA; 5 Department of Human Genetics, University of Chicago, Chicago, IL, USA; 6 Department of Human Molecular Genetics and Biochemistry, Faculty of Medicine, Tel Aviv University, Tel Aviv, Israel; 7 Sheffield Diagnostic Genetic Services, Sheffield Children’s Hospital, Sheffield, UK; 8 School of Clinical Dentistry, University of Sheffield, Sheffield, UK; 9 Princess Máxima Center for Pediatric Oncology, Utrecht, the Netherlands; 10 Office of the Vice Chancellor for Research and Graduate Education, University of Wisconsin-Madison, Madison, WI, USA

**Keywords:** human pluripotent stem cells, genetic changes, culture conditions, MDM4, genome damage

## Abstract

Culture-acquired variants in human pluripotent stem cells (hPSCs) hinder their applications in research and clinic. However, the mechanisms that underpin selection of variants remain unclear. Here, through analysis of comprehensive karyotyping datasets from over 23,000 hPSC cultures of more than 1,500 lines, we explored how culture conditions shape variant selection. Strikingly, we identified an association of chromosome 1q gains with feeder-free cultures and noted a rise in its prevalence in recent years, coinciding with increased usage of feeder-free regimens. Competition experiments of multiple isogenic lines with and without a chromosome 1q gain confirmed that 1q variants have an advantage in feeder-free (E8/vitronectin), but not feeder-based, culture. Mechanistically, we show that overexpression of *MDM4*, located on chromosome 1q, drives variants’ advantage in E8/vitronectin by alleviating genome damage-induced apoptosis, which is lower in feeder-based conditions. Our study explains condition-dependent patterns of hPSC aberrations and offers insights into the mechanisms of variant selection.

## Highlights

• Increase in chromosome 1q gains in recent years is linked to feeder-free cultures
• Feeder-free conditions (E8/vitronectin) confer high levels of genome damage on hPSC
• Amplification of *MDM4* on chromosome 1q alleviates genome damage-induced apoptosis
• Karyotypically abnormal cells have a context-dependent selective advantage

## Graphical abstract

**Figure:**
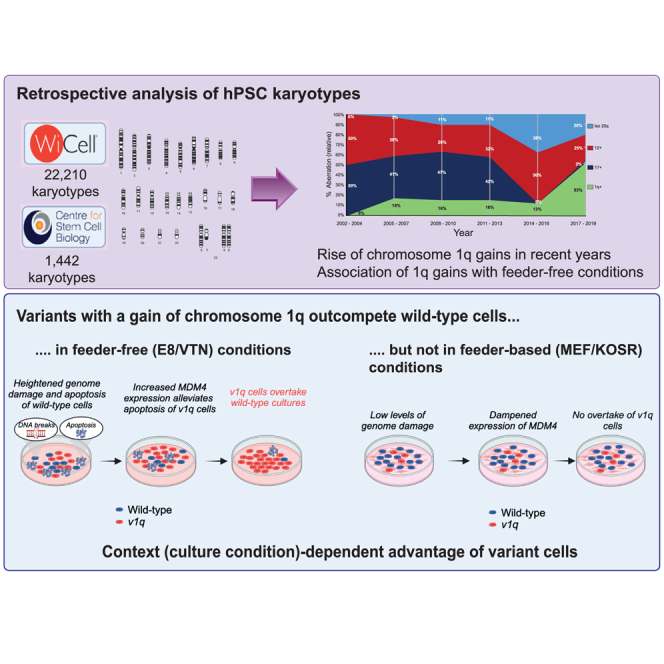

## Teaser

Barbaric and colleagues address a long-standing question of how hPSC culture conditions affect the repertoire of genetic changes. They demonstrate that chromosome 1q gains are associated with feeder-free conditions and explain condition-dependent advantage of variants. Overexpression of *MDM4*, located on chromosome 1q, drives variants’ advantage in feeder-free (E8/vitronectin) conditions by alleviating genome damage-induced apoptosis, which is lower in feeder-based conditions.

## Introduction

The large-scale production of human pluripotent stem cells (hPSCs) for applications in research and medicine necessitates their prolonged maintenance and proliferation in culture. However, *in vitro* expansion predisposes hPSCs to the acquisition of genetic changes, which can impact growth rates, differentiation ability, and tumorigenic potential of hPSCs (Andrews et al., 2017, 2022). It is now well established that genetic changes in hPSCs are non-random, suggesting that recurrent aberrations provide variant cells with selective growth advantage (Halliwell et al., 2020a). However, the mechanisms through which variant hPSCs become fixed in a population remain poorly understood.

The first reported genetic changes in hPSCs were karyotypic abnormalities involving gains of chromosomes 17q and 12 (Draper et al., 2004). Subsequent studies suggested that as many as 35% of hPSC lines become karyotypically abnormal upon prolonged passage (International Stem Cell et al., 2011), with most commonly implicated aberrations involving gains of whole or parts of chromosomes 1, 8, 12, 17, 20, and X and losses of chromosomes 10 and 18 (reviewed in Halliwell et al., 2020a). More recently, other types of aberrations, including sub-karyotypic copy-number variants (CNVs) (such as 20q11.21 [Avery et al., 2013; International Stem Cell et al., 2011]) and single-nucleotide variants (such as variants in the tumor suppressor *TP53* [Lezmi et al., 2024; Merkle et al., 2017]) were also reported.

The culture conditions for hPSCs have changed significantly since they were first derived (Thomson et al., 1998). In the early years, hPSC cultures typically used fibroblast feeder layers and contained serum or KnockOut™ Serum Replacement (KOSR). However, more recently, a variety of feeder-free and KOSR-free culture conditions have become available. Thus, one important question is whether particular genetic changes that occur differ depending on the culture conditions used. Additionally, developing strategies to suppress culture-acquired variants requires identifying the driver genes and the cellular and molecular mechanisms that underlie the growth advantage of variant cells.

In this study we took advantage of a large dataset of hPSC karyotypes collected over a long period through routine monitoring of hPSC samples submitted by diverse groups to WiCell for karyotyping to address whether particular karyotypic changes were affected by changing methods of culture. We found a marked increase in incidence of chromosome 1q gains and isochromosome 20q in recent years, a period associated with a field-wide shift to contemporary, feeder-free/KOSR-free cultures. Focusing on the chromosome 1q gain, we then set out to elucidate the mechanisms through which it confers advantage to cells, identify the driver gene, and explain why this aberration is found mainly in feeder-free, but not feeder-based, conditions.

## Results

### Recurrent karyotypic aberrations in hPSC cultures and changes in their patterns over the last two decades

**Figure 1:**
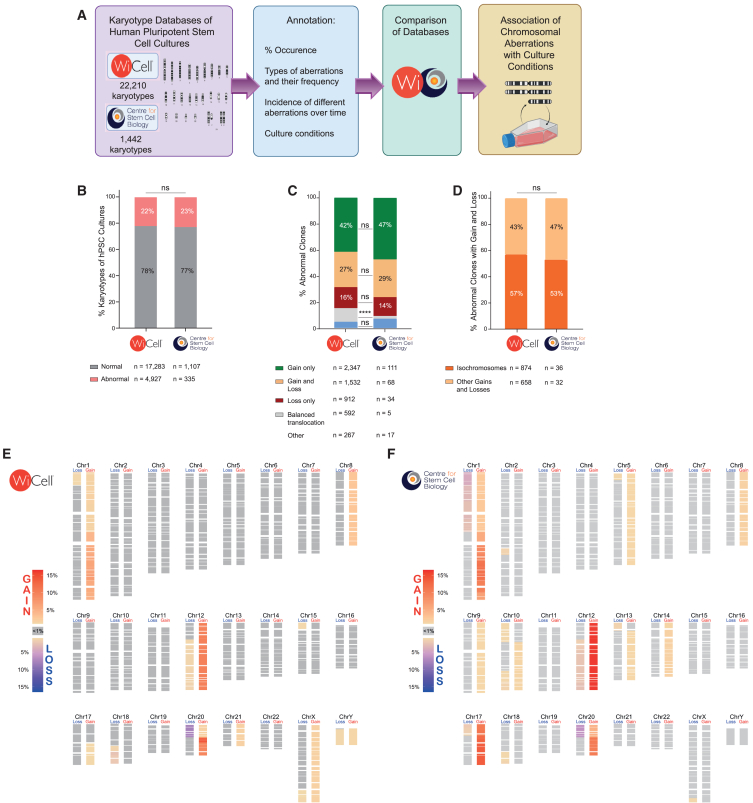
Retrospective analysis of aberrant karyotypes and conditions for culturing hPSCs (A) Karyotyping data from two independent centers (WiCell and Centre for Stem Cell Biology [CSCB]) were annotated for different parameters and analyzed to ascertain a possible association of chromosomal aberrations with culture conditions. (B) Percentage of hPSC cultures containing cells with abnormal karyotypes is similar between WiCell and CSCB datasets. ns, non-significant; Fisher’s exact test. (C) The breakdown of abnormal karyotypes according to the type of abnormality. ns, non-significant; ^∗∗∗∗^*p* < 0.0001; Fisher’s exact test. (D) The majority of aberrations involving both gains and losses of chromosomal material are isochromosomes. ns, non-significant; Fisher’s Exact test. (E) The frequency of gains/losses of each cytoband across all abnormal karyotypes in the WiCell dataset. (F) The frequency of gains/losses of each cytoband across all abnormal karyotypes in the CSCB dataset. See also Tables S1, S2, and Figures S1–S5.

To decipher why we observe recurrent genetic changes in hPSC cultures, we first analyzed karyotypic aberrations from a large dataset of 22,210 hPSC karyotypes acquired by WiCell (www.wicell.org) from samples submitted by many diverse laboratories for routine banking operations between 2009 and 2021. Most of the entries in this database have been annotated with features including the date of the cytogenetic analysis and culture conditions used (Figure 1A; Table S1). In tandem, we also analyzed a smaller in-house dataset from the Centre for Stem Cell Biology (CSCB) in Sheffield, containing 1,442 karyotypes (Figure 1A; Table S1). Whereas the WiCell data reflect cultures from many different labs, though all analyzed at WiCell, the CSCB data reflect data on a much more limited group of cell lines maintained in a single lab. We confirmed the compatibility of the datasets by examining the occurrence and types of abnormalities recorded. In both datasets abnormal clones were present at a similar percentage, i.e., 22% and 23% for WiCell and CSCB, respectively (Figure 1B). Additionally, in both datasets gains of chromosomes or chromosomal regions were the most common karyotypic aberration (Figure 1C). This was followed by occurrences of gains together with losses of chromosomal material within the same karyotype (Figure 1C), the majority of which manifested as isochromosomes (Figure 1D). Aberrations that entailed only the loss of chromosomes or parts of chromosomes were relatively infrequent, as were balanced translocations (Figure 1C). Overall, in line with previous reports (Baker et al., 2016; International Stem Cell et al., 2011; Taapken et al., 2011), these data showed that the appearance of karyotypically abnormal cells is a relatively frequent occurrence in hPSC cultures, with gains of chromosomal materials being more prevalent than losses (Figures 1E, 1F, S1, and S2). Although abnormal cells were seen at a range of passages, a comparison of cultures sampled at different passage numbers showed a clear trend in the proportion of abnormal karyotypes increasing with the increase in passage numbers (Figure S3A), thus reinforcing the importance of minimizing the extent of hPSC expansion in culture (International Stem Cell et al., 2011).

We defined the recurrent abnormalities as aberrations that were present in more than 1% of the total hPSC karyotypes analyzed. While the similarities between the datasets included high frequency of abnormal karyotypes with gains of chromosome 1q (9.3% in WiCell; 14% in CSCB), trisomy 12 (13.7% in WiCell; 20% in CSCB), and isochromosome 20q (7.8% in WiCell; 8.1% in CSCB) (Table S2), we also noted some differences. For instance, 18q loss and trisomy 8 that were frequent in the WiCell dataset (3.9% and 4.6%, respectively) were present in less than 1% of total karyotypes in the CSCB dataset (Table S2). In contrast, the CSCB dataset contained a high frequency of trisomy 17 (14.5%), while in the WiCell data this aberration represented only 0.6% of all abnormalities (Table S2). This discrepancy suggests that there may be differences in culture practices or culture regimens between labs, which may have also changed over time.

The presence of karyotypic abnormalities is thought to predispose cells to genetic instability and further accrual of genetic aberrations (Hanahan and Weinberg, 2011). In cancer, aberrations that commonly appear together in a karyotype have been used to discern functionally important aberrations from changes that represent mere random events (Terragna et al., 2024). Following this logic, we analyzed co-occurrences of different karyotypic aberrations in hPSCs. The total number of karyotypes of clonal aberrations with two or more chromosomes was only 94 in the CSCB dataset, so to address this question we used the larger dataset (WiCell), which had 1,821 clonal aberrations involving two or more chromosomes (8% of the total number). We plotted the percentage of a trisomy of a particular chromosome with another chromosome trisomy/monosomy (Figure S4), and a trisomy of a chromosome with another partial gain/loss of a chromosome (Figure S5). We noted distinct patterns in co-occurrences of some chromosomes. For example, for trisomy 12, which was overall the most common trisomy in the dataset, we noted a co-occurrence mainly with trisomy 8, 14, 17, 20, and X (Figure S4). By analyzing samples that captured mosaic cultures, we could also infer the order of the chromosome appearance. For example, for trisomy 12 with trisomy 14 co-occurrence, in nine instances where mosaic cultures were captured, karyotypes consisted of both clones with trisomy 12 only and clones with trisomy 12 and 14 in the same cell, suggesting that chromosome 12 was the first aberration to occur, followed by chromosome 14 (Table S1). Similarly, of 11 mosaic cultures with co-occurrence of trisomy 12 and 20, in all instances, trisomy 12 was present before trisomy 20 occurred (Table S1). On the other hand, for trisomy 12 co-occurring with trisomy 17, the order of aberrations seemed less stringent as mosaic cultures contained either 12 or 17 as the first aberration (Table S1). Overall, this analysis suggests that a gain of a chromosome does not necessarily make variant hPSCs genetically unstable in a sense that they acquire entirely random genetic changes, but, rather, it suggests that additive or synergistic effects of different aneuploid chromosomes provide selection preferences for variant cells.

**Figure 2:**
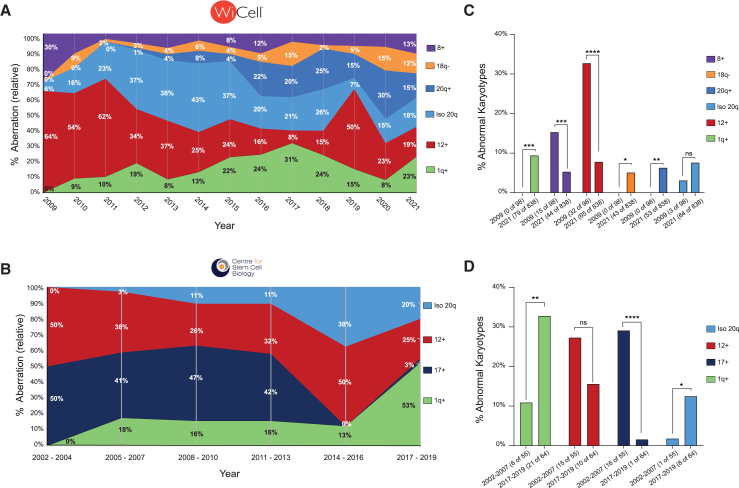
Changes in patterns of recurrent aberrations over time (A) The relative frequency of the six most common abnormalities in the WiCell dataset over time. (B) The relative frequency of the four most common abnormalities in the CSCB dataset over time. (C) The proportion of abnormal karyotypes containing one of the six most common abnormalities in the WiCell dataset sampled in the year 2009 versus the year 2021. ns, non-significant; ^∗^*p* < 0.05, ^∗∗^*p* < 0.01, ^∗∗∗^*p* < 0.001, ^∗∗∗∗^*p* < 0.0001; Fisher’s exact test. (D) The proportion of abnormal karyotypes containing one of the four most common abnormalities in the CSCB dataset sampled in 2002–2007 versus 2017–2019 (several years are pooled together due to relatively low n numbers in this dataset). ns, non-significant; ^∗^*p* < 0.05, ^∗∗^*p* < 0.01, ^∗∗∗∗^*p* < 0.0001; Fisher’s exact test.

Finally, we assessed whether the incidence of the most frequently encountered aberrations in WiCell and CSCB datasets was constant with time. Remarkably, we found a disparity in the representation of different variants over the years (Figures 2A and 2B), with an overall trend toward increase in frequency of aberrations such as gains of chromosome 1q, 20q, and isochromosome 20q at the expense of aberrations such as trisomy 12 and 17 (Figures 2A–2D). In summary, patterns of karyotypic abnormalities have changed over the last two decades, with (iso)chromosome 20q and 1q gains becoming more prevalent—and trisomy 12 and 17 becoming less prevalent—in recent years.

### Increased frequency in chromosome 1q gains associates with usage of KOSR-free culture conditions

**Figure 3:**
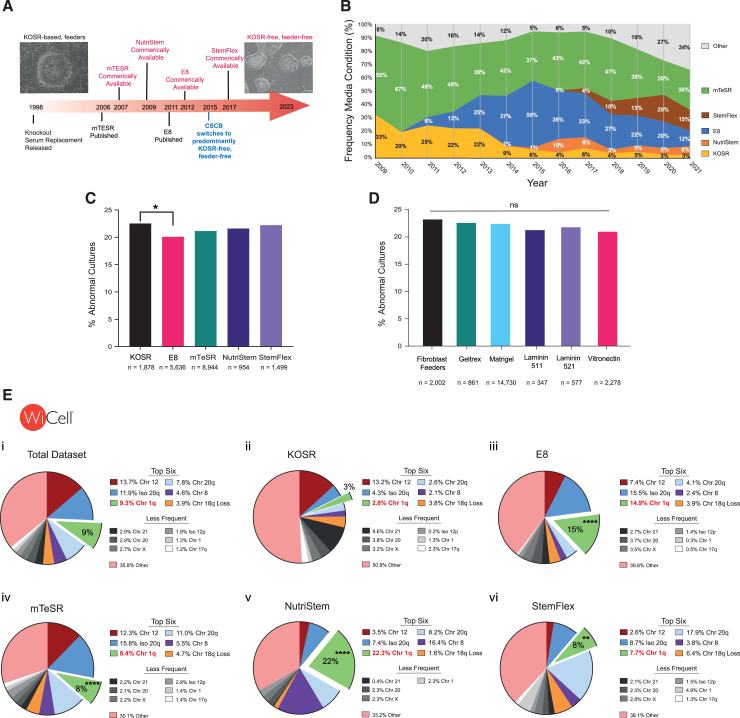
Gains in chromosome 1q are associated with KOSR-free conditions (A) Timeline of the introduction of popular hPSC culture conditions over the past 25 years beginning with predominantly KOSR-based systems and transitioning into KOSR-free culture regimens. CSCB started using predominantly KOSR-free systems in 2015. (B) The relative frequency of the most used media in the WiCell dataset over time. The use of KOSR-based media had decreased over time. (C) The percentage of abnormal karyotypes from the cultures using the most represented media across the WiCell dataset. ^∗^*p* = 0.0253; Fisher’s exact test. (D) The percentage of abnormal karyotypes from the cultures using the most represented matrices across the WiCell dataset. ns, non-significant; Fisher’s exact test. (E) The percentage of the most common abnormalities in the WiCell dataset across (i) all media (5,650 abnormalities), (ii) KOSR-based media (470 abnormalities), (iii) E8 (1,279 abnormalities), (iv) mTESR (2,295 abnormalities), (v) NutriStem (256 abnormalities), and (vi) StemFlex (391 abnormalities). Less frequent but recurrent aberrations (above 1% of abnormal karyotypes) are indicated in shades of gray. In comparison to KOSR-based medium, the frequency of 1q gain is elevated in E8 (^∗∗∗∗^*p* = 7.6 × 10^−15^), mTESR (^∗∗∗∗^*p* = 4.5 × 10^−6^), NutriStem (^∗∗∗∗^*p* = 8.9 × 10^−17^), and StemFlex (^∗∗^*p* = 0.0014); Fisher’s exact test.

Over time, culture conditions have indeed shifted from fibroblast feeders and KOSR-containing media (from herein termed KOSR-based) to a range of feeder-free, KOSR-free regimens (from herein termed KOSR-free) (Figures 3A, 3B, and S3B). We used our annotated karyotyping datasets to assess a potential association of aberrant karyotypes with specific culture conditions. We first assessed whether the percentage of abnormal karyotypes differed significantly depending on the culture medium or matrix used. We found a similar percentage (20%–22%) of abnormal karyotypes in the KOSR-based and the four most represented KOSR-free media across the WiCell dataset (E8, mTeSR, NutriStem, and StemFlex) (Figure 3C). This was the same for the six most used matrices in the WiCell data (feeders, geltrex, matrigel, laminin 511, laminin 521, and vitronectin) (Figure 3D). Overall, this analysis indicated that the overall incidence of karyotypic changes is similar across culture media and matrices. Further, taken together with our findings that the frequencies of different aberrations changed over time (Figure 2), these data suggest that the identity of the chromosome involved in abnormal karyotypes differ based on the medium in which the cells were grown.

Therefore, we next assessed the recurrent abnormality landscape for each of the most represented media in the WiCell dataset and found that the relative frequency of commonly acquired chromosomal aberrations differed across different conditions (Figures 3Ei–vi). Of note, while gains of chromosome 1q represented the third most common abnormality (∼9% of all abnormal karyotypes) across the entire dataset (Figure 3Ei), this aberration was over-represented in the KOSR-free media (15% of all abnormal karyotypes in E8 [*p* = 7.6 × 10^−15^], 8% in mTESR [*p* = 4.5 × 10^−6^] and StemFlex [*p* = 0.0014], and 22% in NutriStem [*p* = 8.9 × 10^−17^]) in comparison to the KOSR-based conditions (3% of all abnormal karyotypes) (Figures 3Eii–vi). A similar trend was also apparent for the isochromosome 20q. The isochromosome 20q represented ∼12% of all abnormal karyotypes in the whole dataset (Figure 3Ei), but it was over-represented in the KOSR-free media (∼16% of all abnormal karyotypes in E8 [*p* = 9.3 × 10^−12^] and mTESR [*p* = 2.6 × 10^−13^] and 9% in StemFlex [*p* = 0.01]) in comparison to the KOSR-based conditions (4% of all abnormal karyotypes) (Figures 3Eii–vi).

Together, our analysis revealed that the landscape of common abnormalities in hPSCs had changed over time, seemingly coinciding with changes in culture conditions utilized. Specifically, we detected an increase in the prevalence of isochromosome 20q and gains of chromosome 1q in association with the usage of KOSR-free conditions in recent years.

### Variant hPSCs with a gain of chromosome 1q show selective advantage in a context-dependent manner

**Figure 4:**
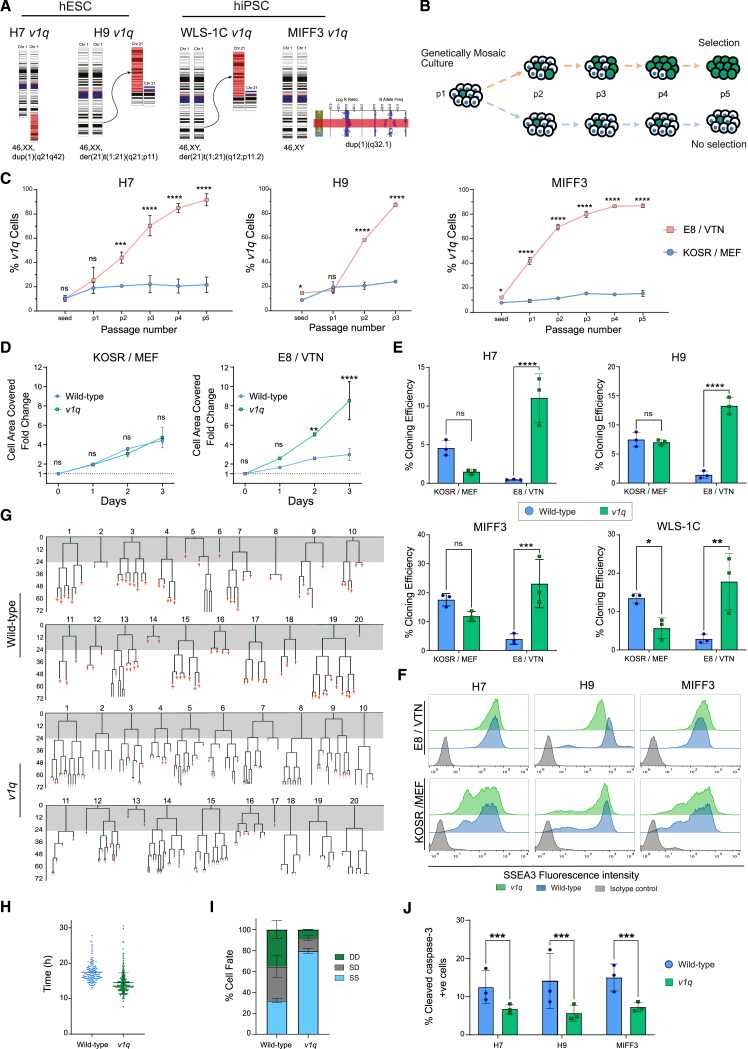
*v1q* have selective advantage in KOSR-free but not KOSR-based conditions (A) A panel of wild-type and *v1q* sublines across four genetic backgrounds (H7, H9, MIFF3, and WLS-1C) used in this study. (B) Selective advantage was tested by mixing ∼10% *v1q* with their wild-type counterparts, with either of the lines being fluorescently labeled. Mixed cells were plated into either KOSR/MEF or E8/VTN, and the ratio of variants was monitored over subsequent passages. (C) *v1q* overtake wild-type cells rapidly in E8/VTN but not in KOSR/MEF. Data shown are the mean ± SD of three independent experiments. ns, non-significant; ^∗^*p* < 0.05, ^∗∗^*p* < 0.01, ^∗∗∗^*p* < 0.001; ^∗∗∗∗^*p* < 0.0001; two-way ANOVA. (D) *v1q* have a significantly higher growth rate than wild-type cells in E8/VTN but not KOSR/MEF. Data shown are the mean ± SD of three independent experiments. ns, non-significant; ^∗∗^*p* < 0.01, ^∗∗∗∗^*p* < 0.0001; two-way ANOVA. (E) *v1q* have a significantly higher cloning efficiency than wild-type cells in E8/VTN but not KOSR/MEF. Data shown are the mean ± SD of three independent experiments. ns, non-significant; ^∗^*p* < 0.05, ^∗∗^*p* < 0.01, ^∗∗∗^*p* < 0.001, ^∗∗∗∗^*p* < 0.0001. two-way ANOVA, Fisher’s least significant difference. (F) Wild-type and *v1q* hPSCs display similar levels of expression of a marker of undifferentiated state, SSEA3, in E8/VTN and in KOSR/MEF conditions. (G) Lineage trees tracked from time-lapse images of wild-type cells (upper two panels) and *v1q* (lower two panels). Red crosses indicate cell death. Gray-shaded area indicates the first 24 h post-plating when the cells were grown in the presence of Y-27632, required for single-cell passaging. (H) *v1q* show a trend toward a faster cell-cycle time compared to wild-type counterparts. Data points indicate 114 and 286 divisions for wild-type and *v1q* cells, respectively, from two independent experiments. (I) Percentage of cell fate outcomes of daughter cells following cell division, with SS denoting survival of both daughter cells, SD survival of one and death of the other daughter cell, and DD death of both daughter cells. (J) *v1q* have decreased levels of cleaved caspase-3 marker of apoptosis. Data shown are the mean ± SD of three independent experiments. ^∗∗∗^*p* < 0.001; two-way ANOVA followed by Holm-Sidak’s multiple comparison test. See also Video S1 and Figure S6.

Focusing on chromosome 1q gains, we next set out to unravel the mechanistic basis for the apparent differences in their prevalence under different culture conditions. The increased frequency of chromosome 1q gains among karyotypes of hPSCs grown in KOSR-free conditions could be a consequence of 1q variant cells possessing the selective advantage in KOSR-free, but not KOSR-based, conditions. To test this hypothesis, we utilized pairs of cells with a gain of a portion of chromosome 1q (from herein *v1q*) and their isogenic wild-type counterparts across several different genetic backgrounds: H7 (WA07) (Thomson et al., 1998), H9 (WA09) (Thomson et al., 1998), MIFF3 (Desmarais et al., 2016), and WLS-1C (Chang et al., 2013) (Figures 4A, S6A, and S6B).

We first performed competition experiments in which we mixed a small proportion (∼10%) of *v1q* cells into wild-type cultures and then monitored the ratio of the two sublines over subsequent passages (Price et al., 2021) either in KOSR-based medium on mouse embryonic fibroblasts (KOSR/MEF) or in KOSR-free E8/vitronectin (E8/VTN) conditions (Figure 4B). Strikingly, while, in KOSR-based conditions, the ratio of *v1q* overall remained unchanged over several consecutive passages, in E8/VTN *v1q* rapidly overtook the cultures, and this result was consistent across all three pairs of lines (H7, H9, and MIFF3) tested (Figure 4C). *v1q* cells also displayed faster population growth in comparison to wild-type cells only in E8/VTN, but not in KOSR/MEF, condition (Figure 4D). Finally, we utilized a clonogenic assay as a particularly sensitive test of the ability of individual hPSCs to survive replating and initiate stem cell colonies (Barbaric et al., 2014). The clonogenic assay showed an increased cloning ability of *v1q* compared to wild-type hPSCs, again only when cloning was performed in E8/VTN, but not KOSR/MEF, conditions (Figure 4E). Overall, these data support the hypothesis that *v1q* hPSCs have a selective advantage over wild-type hPSCs in E8/VTN, but not KOSR-based, cultures. Importantly, we found no evidence of differences in the levels of spontaneous differentiation between paired wild-type cells and *v1q* (Figures 4F and S6C), suggesting that differences in survival and/or proliferation, rather than differences in the level of spontaneous differentiation, underpin the selective advantage of *v1q*.

We next assessed the reasons behind improved proliferation rates of *v1q* cells in E8/VTN condition. The time-lapse analysis of single cells in E8/VTN revealed a shorter cell-cycle time of *v1q* (Figures 4G and 4H; Video S1) and their improved survival, as fewer numbers of *v1q* underwent cell death upon replating and following cell division compared to their wild-type counterparts (Figures 4G–4I). Consistent with these findings, a lower proportion of cells in *v1q* cultures displayed the cleaved caspase-3 marker of apoptosis (Figure 4J). Together, these results reveal that increased proliferation and reduced apoptosis of *v1q* cells underscore their selective advantage in E8/VTN condition.

Video S1. Time-lapse video of H7 (left) and H7 *v1q* cells (right) grown in E8/VTN, related to Figure 4 Images were taken every 10 min over 96 h.

### MDM4 drives the selective advantage of 1q variant hPSCs in E8/VTN condition

**Figure 5:**
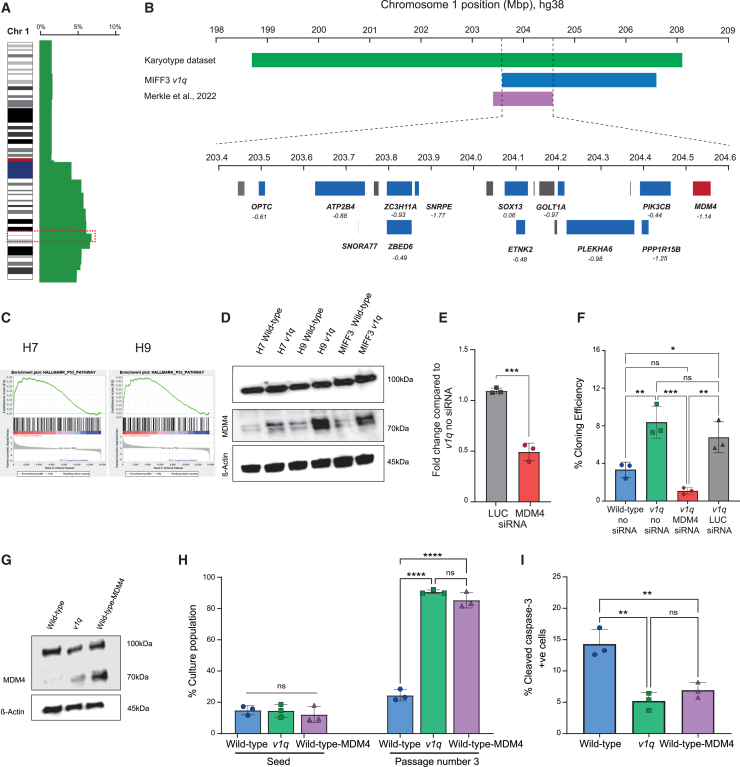
MDM4 overexpression provides selective advantage to *v1q* (A) The minimal region on chromosome 1q32.1 identified from the karyotyping datasets in this study. (B) The minimal region on chromosome 1q32.1 identified from overlaying the karyotyping datasets in this study, SNP array data from MIFF3 *v1q* used in this study, and data published by (Merkle et al., 2022). The minimal amplicon contains 13 genes expressed in hPSCs (blue boxes), which were compared based on their essentiality scores (indicated in italics underneath the genes; for comparison, an essentiality score for *POU5F1* is −1.85). *MDM4* (red) is a candidate of interest, based on its known role in p53 signaling and cancer. (C) RNA-seq analysis of H7 and H9 *v1q* versus wild-type sublines revealed differential expression of the p53 pathway. (D) MDM4 expression is increased in *v1q*. Western blot analysis of H7, H9, and MIFF3 wild-type and v1q cells. β-actin was used as a loading control. (E) Knockdown of MDM4 with small interfering RNA (siRNA) in *v1q* was confirmed by quantitative PCR. siRNA for Renilla Luciferase (siRNA LUC) was used as a negative control. Data shown are the mean ± SD of three independent experiments. ^∗∗∗^*p* < 0.001; Student’s t test. (F) MDM4 knockdown suppresses cloning efficiency of *v1q*. Data shown are the mean ± SD of three independent experiments. ns, non-significant, ^∗^*p* < 0.05, ^∗∗^*p* < 0.01, ^∗∗∗^*p* < 0.001; One-way ANOVA followed by Holm-Sidak’s multiple comparison test. (G) Overexpression of MDM4 in wild-type hPSCs. Western blot analysis of MIFF3, MIFF3 *v1q*, and MIFF3 wild-type cells overexpressing MDM4 (wild-type-MDM4). β-actin was used as a loading control. (H) MDM4 overexpression provides selective advantage to wild-type hPSCs. Mixed cultures were analyzed at seeding and after three passages. Data shown are the mean ± SD of three independent experiments. ns, non-significant, ^∗∗∗∗^*p* < 0.001; One-way ANOVA followed by Holm-Sidak’s multiple comparison test. (I) MDM4 overexpressing and *v1q* cells have lower percentage of cleaved caspase-3 marker of apoptosis compared to wild-type counterparts. Data shown are the mean ± SD of three independent experiments. ns, non-significant, ^∗∗^*p* < 0.01; one-way ANOVA followed by Holm-Sidak’s multiple comparison test.

To identify potential driver genes underpinning the selective advantage of a gain of chromosome 1q in E8/VTN, we mapped the minimal region amplified among all the *v1q* in the karyotyping datasets (Figure 5A). Further, overlaying our SNP array data with a previously reported 1q minimal amplicon in hPSCs (Merkle et al., 2022) enabled us to narrow the candidate region on chromosome 1q32.1 to ∼1 Mb, encompassing 13 genes expressed in hPSCs (Figure 5B). We compared the expressed genes based on their essentiality to the pluripotent state (Yilmaz et al., 2018) (Figure 5B). Notably, one of the top three most essential genes within this region is *MDM4*, a known regulator of TP53 (Karni-Schmidt et al., 2016) and a putative driver gene in the pathology of multiple types of cancers (Girish et al., 2023; Hullein et al., 2019). RNA sequencing (RNA-seq) analysis of H7 and H9 *v1q* versus wild-type sublines further revealed differential expression of the p53 pathway in the *v1q* hPSCs (Figure 5C). Thus, we posited that the increase in copy number of *MDM4* due to the amplification of chromosome 1q32 could provide selective advantage of *v1q* in E8/VTN conditions.

Indeed, in E8/VTN conditions, *v1q* showed higher abundance of MDM4 protein expression, consistent with an increased copy number of *MDM4* (Figure 5D). To functionally probe the role of MDM4 in hPSC selective advantage, we knocked down MDM4 in *v1q* cells (Figure 5E), which resulted in significant decrease in their cloning ability in E8/VTN conditions (Figure 5F). Conversely, we overexpressed MDM4 in wild-type hPSCs and found that increase in MDM4 (Figure 5G) phenocopied the selective advantage of *v1q*, as MDM4-overexpressing cells outcompeted wild-type cells in competition experiments, to the same extent as *v1q* did (Figure 5H). Moreover, MDM4-overexpressing cells exhibited lower levels of cleaved caspase-3 compared to wild-type cells (and similar levels to those of *v1q*; Figure 5I), consistent with the acquisition of an anti-apoptotic phenotype due to MDM4 overexpression. Overall, these data demonstrate that MDM4 amplification is a key contributor to the selective advantage of *v1q* in E8/VTN conditions.

### Lower levels of genome damage and dampened MDM4 expression suppress growth advantage of variant 1q cells in KOSR/MEF

**Figure 6:**
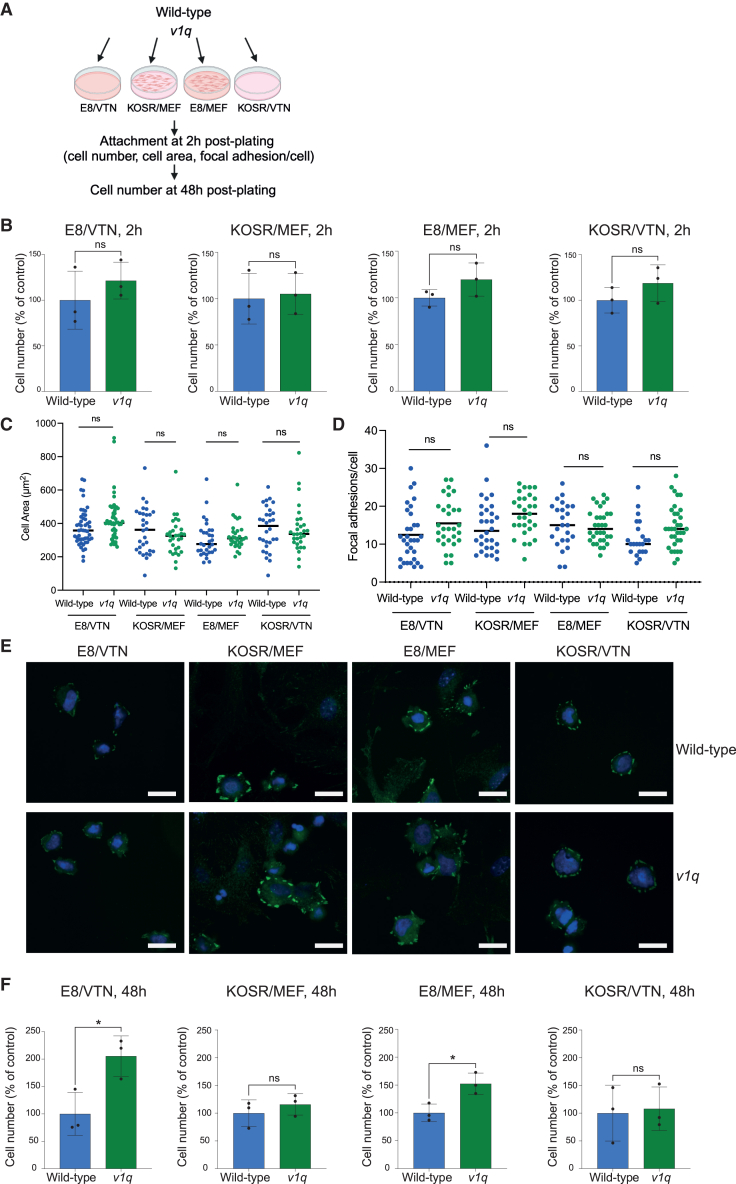
KOSR-based medium, rather than MEFs, diminishes advantage of *v1q* cells in KOSR/MEF compared to E8/VTN (A) Schematic representation of experiments aimed at revealing the impact of matrix (VTN or MEFs) versus medium (E8 or KOSR) on the selective advantage of *v1q*. (B) Similar numbers of MIFF3 wild-type and *v1q* hPSCs attach regardless of the medium (E8 and KOSR) or the matrix (VTN and MEFs) at 2 h post-plating. Data shown are the mean ± SD of three independent experiments. ns, non-significant; Unpaired t test. (C) Cell area at 2 h post-plating is similar between MIFF3 wild-type and *v1q* hPSCs in each of the conditions tested, indicating similar ability of cell to attach post-plating. Circles represent the cell area of individual cells measured at 2 h post-plating. For each test condition, 30–50 cells were measured across three independent experiments. The line represents the mean of all measurements. ns, non-significant, Mann-Whitney test. (D) Quantification of the number of phosphorylated focal adhesion kinase (pFAK)-positive focal adhesions. Circles represent the cell area of individual cells measured at 2 h post-plating. For each test condition, 21–32 cells were measured across three independent experiments. The line represents the mean of all measurements. ns, non-significant, Mann-Whitney test. (E) Representative images of pFAK (green) in MIFF3 wild-type and *v1q* hPSCs plated in different media (E8 and KOSR) or matrices (VTN and MEFs) at 2 h post-plating. Nuclei are counterstained with Hoechst 33342. Scale bar: 25 μm. (F) Numbers of MIFF3 *v1q* hPSCs are higher than wild-type cells in E8/VTN but are not significantly different from wild-type cells in other test conditions at 48 h post-plating. ns, non-significant, ^∗^*p* < 0.05; Unpaired t test. See also Figures S7A and S7B.

Why is the MDM4-mediated 1q gain effect specific to the E8/VTN condition? It is plausible that *v1q* cells attach poorly to MEFs upon replating or that a component of KOSR-based medium may be affecting *v1q* growth, or a combination of both. We plated wild-type and *v1q* cells in different combinations of matrix (VTN, MEF) and media (E8, KOSR) (Figure 6A). Similar numbers of *v1q* cells attached to VTN and MEFs in either E8- or KOSR-based media (Figures 6B and S7A). Moreover, cell area (Figure 6C) and the number of phosphorylated focal adhesion kinase (pFAK)-positive focal adhesions (Figures 6D and 6E) at 2 h post-plating were also similar for wild-type and *v1q* cells, suggesting an equivalent ability of wild-type and *v1q* cells to adhere to the matrix. However, at 48 h post-plating, the number of *v1q* cells surpassed the wild-type numbers when cells were grown on VTN or MEF in E8, but not on VTN or MEF in KOSR-based medium (Figures 6F and S7B). These results suggested that the initial attachment to MEFs is not disadvantaging *v1q* cells per se but, rather, that the KOSR-based medium is suppressing their subsequent proliferation.

**Figure 7:**
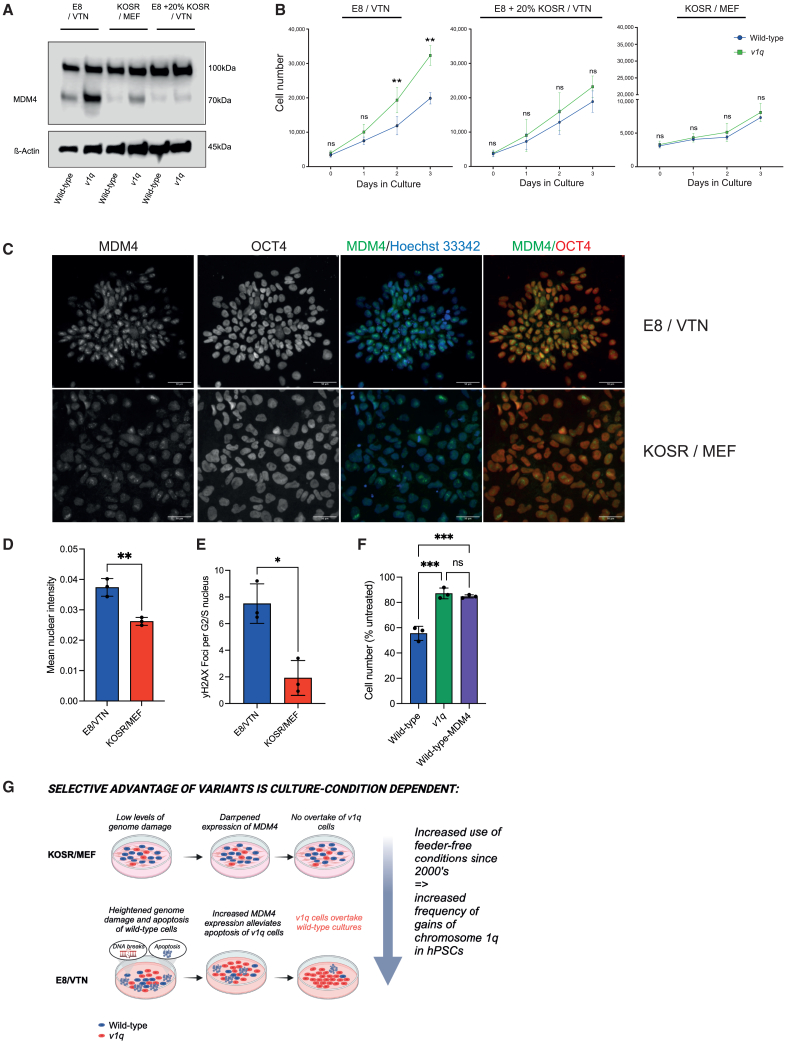
Variant 1q and MDM4-overexpressing cells are less sensitive to DNA double-strand breaks, which are more abundant in hPSCs grown in E8/VTN compared to KOSR/MEF (A) Western blot analysis of MDM4 in MIFF3 and MIFF3 *v1q* cells under different conditions. MDM4 abundance is reduced in KOSR/MEF. Also, the addition of KOSR to E8/VTN reduces MDM4 expression. β-actin was used as a loading control. (B) The addition of KOSR to E8/VTN reduces growth rates of *v1q* to levels like those of wild-type cells. Data shown are the mean ± SD of three independent experiments. ns, non-significant; ^∗∗^*p* < 0.01; two-way ANOVA with Holm-Sidak’s multiple comparison test. (C) MDM4 has a more nuclear and punctate localization in hPSCs grown in E8/VTN compared to KOSR/MEF. Representative images of MIFF3 hPSCs grown in E8/VTN (upper panels) or KOSR/MEF (lower panels) stained with antibodies against MDM4 and OCT4 (POU5F1). The nuclei are counterstained with Hoechst 33342. Scale bar: 50 μm. (D) Quantification of the MDM4 nuclear expression in E8/VTN versus KOSR/MEF condition in MIFF3 line. Data shown are the mean ± SD of three independent experiments. ^∗∗^*p* < 0.01; Unpaired t test. (E) Genome damage is heightened in E8/VTN compared to KOSR/MEF condition. Quantification of γH2AX marker of double-strand breaks in hPSCs grown in E8/VTN compared to KOSR/MEF. Data shown are the mean ± SD of three independent experiments. ^∗^*p* < 0.05; paired t test. (F) Cells with a gain of chromosome 1q or overexpressing MDM4 are more resistant to genome damage-induced apoptosis. Quantification of cell numbers of wild-type, *v1q* and wild-type-MDM4 cells treated with 10 μM camptothecin (CPT) for 2 h. Data shown are normalized to untreated control and represent the mean ± SD of three independent experiments. ^∗∗∗^*p* < 0.001; One-way ANOVA. (G) A model summarizing a differential competitive advantage of *v1q* in E8/VTN versus KOSR/MEF conditions. E8/VTN confers high levels of genome damage in hPSCs. Amplification of MDM4 through the gain of chromosome 1q bestows *v1q* cells with the resistance to genome damage-induced cell death. Consequently, *v1q* outcompete wild-type hPSCs in E8/VTN. The KOSR/MEF condition does not generate the same selective pressure as the levels of genome damage are reduced compared to E8/VTN. The shift from feeder-based to feeder-free conditions over the last two decades has contributed to an increase in frequency of chromosome 1q gains detected in hPSC cultures. See also Figures S7C–S7E.

As we established that MDM4 is driving advantage of *v1q* cells (Figure 5), we next turned to investigating the possibility that the E8- and KOSR-based media differentially affect *v1q* cells by affecting MDM4 expression. In the KOSR/MEF condition, we noted reduced expression of MDM4 in the *v1q* cells compared to E8/VTN conditions (Figure 7A). Remarkably, the addition of KOSR to E8/VTN cultures abolished MDM4 expression (Figure 7A) and suppressed proliferation rates of *v1q* over several days in culture (Figure 7B). Apart from differences in the level of MDM4 expression, we also noted differences in MDM4 localization in E8/VTN versus KOSR/MEF condition. Specifically, we saw punctate nuclear expression of MDM4 in cells grown in E8/VTN, in contrast to a more even distribution of MDM4 throughout the cytoplasm and nucleus of cells grown in KOSR/MEF condition (Figures 7C, 7D, S7C, and S7D).

The nuclear expression of MDM4 was previously associated with its role in response to genome damage (LeBron et al., 2006), and, accordingly, we also found that the induction of genome damage in hPSCs causes nuclear localization of MDM4 (Figure S7E). These observations suggested that the differences we observed in MDM4 localization in E8/VTN versus KOSR/MEF may be due to different levels of genome damage in these two culture regimens. Indeed, in line with previously published studies (Prakash Bangalore et al., 2017), we saw increased levels of γH2AX marker of double-strand breaks in cells grown in E8/VTN compared to KOSR/MEF (Figure 7E). Thus, we surmised that the increased MDM4 expression may enable better survival of cells in conditions of high genome damage. We next deliberately induced double-strand breaks in wild-type, *v1q*, and wild-type-MDM4-overexpressing cells using a topoisomerase I inhibitor camptothecin (CPT) and assessed cell survival following the CPT treatment. We saw higher survival of *v1q* and wild-type-MDM4-overexpressing cells compared to wild-type counterparts upon CPT treatment (Figure 7F), supporting the hypothesis that MDM4 overexpression confers resistance to cell death upon genome damage.

Overall, our data show that variant cells have context-dependent advantage, with high levels of genome damage in E8/VTN favoring the selection of *v1q* cells. The KOSR/MEF conditions reduce both genome damage and MDM4 protein expression, thereby drastically attenuating the selective advantage of *v1q* hPSCs (Figure 7G). A clear implication of these findings is that the recurrent genetic changes observed in hPSCs are directly related to the specific selection pressures conferred by their culture regimens.

## Discussion

Our study directly addresses the long-standing question of whether and how hPSC culture conditions affect the selection of specific genetic changes. Tracking and correlating particular abnormalities with culture regimens are difficult in a classic laboratory setting due to low sample numbers and a narrow range of conditions typically assessed. Our ability to interrogate retrospectively a large karyotyping dataset, sampled across two decades from multiple sources and conditions, allowed us to identify trends in culture-acquired genetic changes that may be linked to changes in cell culture practice, thus providing clues to the possible mechanisms underlying the selective growth advantages of particular changes.

As genetic testing of hPSCs has traditionally relied on cytogenetic methods, such as karyotyping by G-banding, karyotyping datasets used in our study span many years and contain extensive numbers of hPSC samples. However, the resolution of karyotyping by G-banding is relatively limited compared to newer molecular techniques, such as SNP arrays or next-generation sequencing (Andrews et al., 2022; Baker et al., 2016). For instance, G-banding typically fails to detect aberrations that are smaller than 5 Mb and cannot detect sequence changes. This means that some of the recurrent hPSC aberrations, such as chromosome 20q that is typically acquired as a CNV (International Stem Cell et al., 2011), may be underrepresented in the datasets we analyzed. Thus, further analysis of samples assessed by higher-resolution techniques is warranted in the future to assess potential new sub-karyotypic aberrations or changes in their trends in association with culture conditions. Nonetheless, from our analysis we identified the possibility that gains of the long arm of chromosome 1, which have become more prevalent in recent years, provide a selective advantage in the newer feeder-free culture conditions, a hypothesis that we were able to validate directly by comparing the growth behavior of isogenic pairs of lines with and without a gain for chromosome 1q.

To identify a driver gene on chromosome 1q, we focused on *MDM4*, which is located in the minimally amplified region. This gene has been previously shown to be an inhibitor of *TP53*, which is the most growth-restricting (Yilmaz et al., 2018) and also the most frequently mutated gene in hPSCs (Avior et al., 2021; Lezmi et al., 2024), attesting to the importance of p53 in hPSC biology and suggesting that regulators of p53, such as MDM4, may also be a key target of genetic changes. Here, we showed that the forced expression of MDM4 in wild-type hPSCs phenocopies *v1q* suggesting that *MDM4* is a likely driver gene for this amplification in hPSCs. Intriguingly, chromosome 1q is amplified in many cancers (Karni-Schmidt et al., 2016) and *MDM4* was recently proposed as a driver of recurrent chromosome 1q gains in breast cancer through increased expression of MDM4 and reduced TP53 signaling (Girish et al., 2023). Nonetheless, it is important to note that additional genes present in the amplified region of chromosome 1q could also play a role in the behavior of variant cells or their differentiated derivatives.

Similarities in the percentage of abnormal karyotypes across different conditions inferred from our karyotyping dataset suggest that commonly used conditions give rise to a similar rate of karyotypic abnormalities. Thus, based on these data we cannot advocate specific matrix/media combinations for growing hPSCs. However, the differences in the identity of chromosomes implicated in different culture conditions suggest that different selective pressures may operate across different culture conditions. Therefore, it stands to reason that identifying specific selective pressures in each of the conditions may ultimately provide a way of designing culture conditions in which key selective pressures for growing hPSCs are alleviated. In this study we have particularly shown that culture of hPSCs in KOSR-free conditions (E8/VTN) results in a greater susceptibility to genomic damage than growth in the presence of KOSR and feeders, which can be prevented by overexpression of MDM4 resulting from gains of chromosome 1q.

Previous work by us and others in the field demonstrated that, unlike somatic cells, hPSCs respond to the induction of genome damage predominantly by committing to apoptosis (Desmarais et al., 2012; Halliwell et al., 2020b). Such a reliance of hPSCs on apoptosis for clearing the genetically damaged cells may render them highly vulnerable to specific genetic changes that prevent the apoptotic response to genome damage (Halliwell et al., 2020a). Of note, another anti-apoptotic gene, *BCL2L1*, has been previously identified as the driver gene behind the 20q11.21 amplification. The identification of recurrent mutations in *TP53* (Lezmi et al., 2024; Merkle et al., 2017) further supports the view that recurrent aberrations in hPSCs converge on an anti-apoptotic phenotype. Nonetheless, it remains to be identified whether other aberrations frequently detected in hPSC culture, such as isochromosome 20q or chromosome 18q loss, also alleviate genome damage-induced cell death or whether their preponderance in culture signifies the presence of alternative or additional selective pressures. Further collation of well-annotated genetic datasets, complemented by prospective studies of variant emergence and behavior in different culture regimens, will be required to unequivocally address this outstanding question. Nonetheless, most of the abnormalities noted in routine hPSC karyotyping are not reported in the literature, making up-to-date findings on abnormalities inaccessible to the community. Therefore, it has been proposed that a community-shared database should be created in order to collate genetic changes in hPSCs to enable timely identification of links between culture practices and genetic aberrations (Andrews et al., 2022). A database collated in this study provides a first step toward that goal.

Overall, our discoveries provide the mechanistic understanding for differences in the recurrent genetic changes in hPSCs grown under different culture conditions and set the stage for targeted strategies for minimizing the appearance of recurrent aberrations. Moreover, as gains of chromosome 1q are common in many different types of cancer, demonstration of the principles behind the context-dependent advantage of variants may have broader implications for understanding and tackling selective advantage of cancerous cells. Finally, this study also highlights the usefulness of using variant hPSCs to gain a mechanistic understanding into what controls hPSC behavior in different signaling environments and in a broader sense how context-dependent advantages of genetic variants of cancer cells could be negated.

## Experimental procedures

### Resource availability

#### Lead contact

Further information and requests for resources and reagents should be directed to and will be fulfilled by the lead contact Ivana Barbaric (i.barbaric@sheffield.ac.uk).

#### Materials availability

Materials generated in this study are available from the lead contact upon request.

#### Data and code availability

The RNA-seq data have been deposited to ArrayExpress; accession number E-MTAB-13383.

### Karyotyping database assembly and analyses

Karyotyping data analyzed in this study were collected by WiCell (Madison, USA) and the Centre for Stem Cell Biology (CSCB) (Sheffield, UK) over a period of 2009–2021 and 2002–2019, respectively. While CSCB data were mainly generated from hPSC lines grown in-house (around 80 different lines), WiCell data contained hPSC samples that were not only grown in-house but also submitted to WiCell for cytogenetic analysis by different laboratories (estimated to contain over 1,500 different cell lines). For details on data curation and analysis, see supplemental experimental procedures.

To facilitate further exploration of our collated dataset, we also compiled a browser-based database termed KaryoBrowser (https://karyo.group.shef.ac.uk/). KaryoBrowser allows filtering of dataset based on different parameters, including culture media, matrix, whether the lines are embryonic or induced PSCs, and their sex. The filtered data containing the karyotypes can also be exported for further analysis.

### hPSC lines

Wild-type hPSCs used in the experimental part of this study were H7 (WA07) (Thomson et al., 1998) (RRID:CVCL_9772), H9 (WA09) (Thomson et al., 1998) (CVCL_9773), MIFF3 (Desmarais et al., 2016) (CVCL_1E70), and WLS-1C (Chang et al., 2013). Wild-type sublines were karyotypically normal (based on at least 20 metaphases analyzed by G-banding of cell banks prior to experiments). Genetically variant hPSCs used in this study and their karyotypes were H7 *v1q* (RRID:CVCL_A5KR; [46,XX,dup(1)(q21q42)]), H9 *v1q* [46,XX,der(21)t(1; 21)(q21; p11)], MIFF3 *v1q* [46,XY], and WLS-1C *v1q* [46,XY,der(21)t(1; 21)(q12; p11.2)]. See also supplemental experimental procedures.

### hPSC culture

See supplemental experimental procedures.

### Karyotyping by G-banding and SNP arrays

See supplemental experimental procedures.

### Competition assays

Competition experiments were performed using matched fluorescently labeled and unlabeled lines, as follows: H7 *v1q* with H7-RFP cells, H9 *v1q* with H9-RFP cells, MIFF3 *v1q*-H2B-GFP with MIFF3 counterparts, and MIFF3-GFP cells with MIFF3 *v1q* or MIFF3-MDM4 cells. See also supplemental experimental procedures.

### MDM4 small interfering RNA knockdown and overexpression

To knock down the expression level of MDM4 in MIFF3 *v1q* cells, we used MISSION esiRNA for MDM4 (ESIRNA HUMAN MDMX, Cat. # EHU005381-20UG; Sigma-Aldrich) and MISSION esiRNA for Renilla luciferase as a control (ESIRNA RLUC Cat. # EHURLUC-20UG; Sigma-Aldrich). See also supplemental experimental procedures.

For MDM4 overexpression, the pCAG-MDM4 expression vector was established by cloning of MDM4 sequence into a pCAG vector (Liew et al., 2007). To generate the MIFF3 wild-type MDM4-overexpressing line, cells were transfected using the Neon transfection system. See also supplemental experimental procedures.

### Quantification and statistical analysis

Statistical analysis of the data presented was performed using either GraphPad Prism version 9.0.2, GraphPad Software, La Jolla California, USA (www.graphpad.com), or Real Statistics Resource Pack for Excel, Charles Zaiontz (www.real-statistics.com). Differences were tested by statistical tests as indicated in the figure legends.

## Supplemental information

Document S1. Figures S1–S7 and supplemental experimental procedures

Table S1. Karyotyping datasets from WiCell and CSCB, related to Figure 1

Table S2. CytoGPS analysis of frequencies at which cytobands are represented in abnormal hPSC karyotypes in the WiCell and CSCB dataset, related to Figure 1 and Figures S1 and S2 Karyotypic abnormalities as percentage of abnormal and total karyotypes in the WiCell and CSCB dataset.

Document S2. Article plus supplemental information
