# Supplementary material for: Feeder-free culture of human pluripotent stem cells drives MDM4-mediated gain of chromosome 1q
Source: Stem Cell Reports. 2024 Jul 3;19(8):1217–32. doi: 10.1016/j.stemcr.2024.06.003 (PMC11368687; doi:10.1016/j.stemcr.2024.06.003)
Supplement: Document S1. Figures S1–S7 and supplemental experimental procedures [file mmc1.pdf]

Stem Cell Reports, Volume *19*

Supplemental Information

Feeder-free culture of human pluripotent stem cells drives MDM4-mediated gain of chromosome 1q

Dylan Stavish, Christopher J. Price, Gabriele Gelezauskaite, Haneen Alsehli, Kimberly A. Leonhard, Seth M. Taapken, Erik M. McIntire, Owen Laing, Bethany M. James, Jack J. Riley, Johanna Zerbib, Duncan Baker, Amy L. Harding, Lydia H. Jestice, Thomas F. Eleveld, Ad J.M. Gillis, Sanne Hillenius, Leendert H.J. Looijenga, Paul J. Gokhale, Uri Ben-David, Tenneille E. Ludwig, and Ivana Barbaric

Figure S1

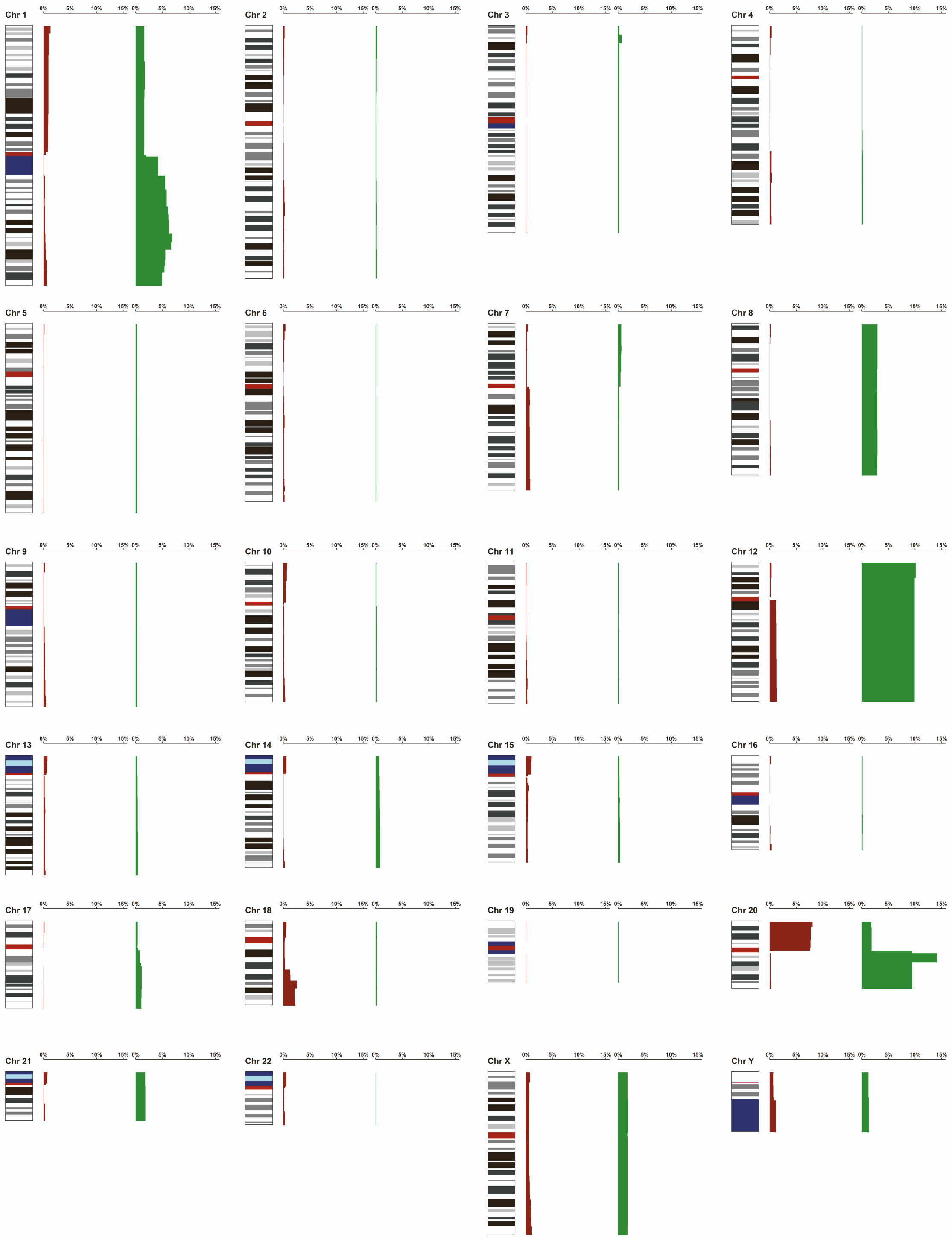
Chr 1
Chr 2
Chr 3
Chr 4
Chr 5
Chr 6
Chr 7
Chr 8
Chr 9
Chr 10
Chr 11
Chr 12
Chr 13
Chr 14
Chr 15
Chr 16
Chr 17
Chr 18
Chr 19
Chr 20
Chr 21
Chr 22
Chr X
Chr Y
0%
5%
10%
15%

Centre for Stem Cell Biology

Figure S2

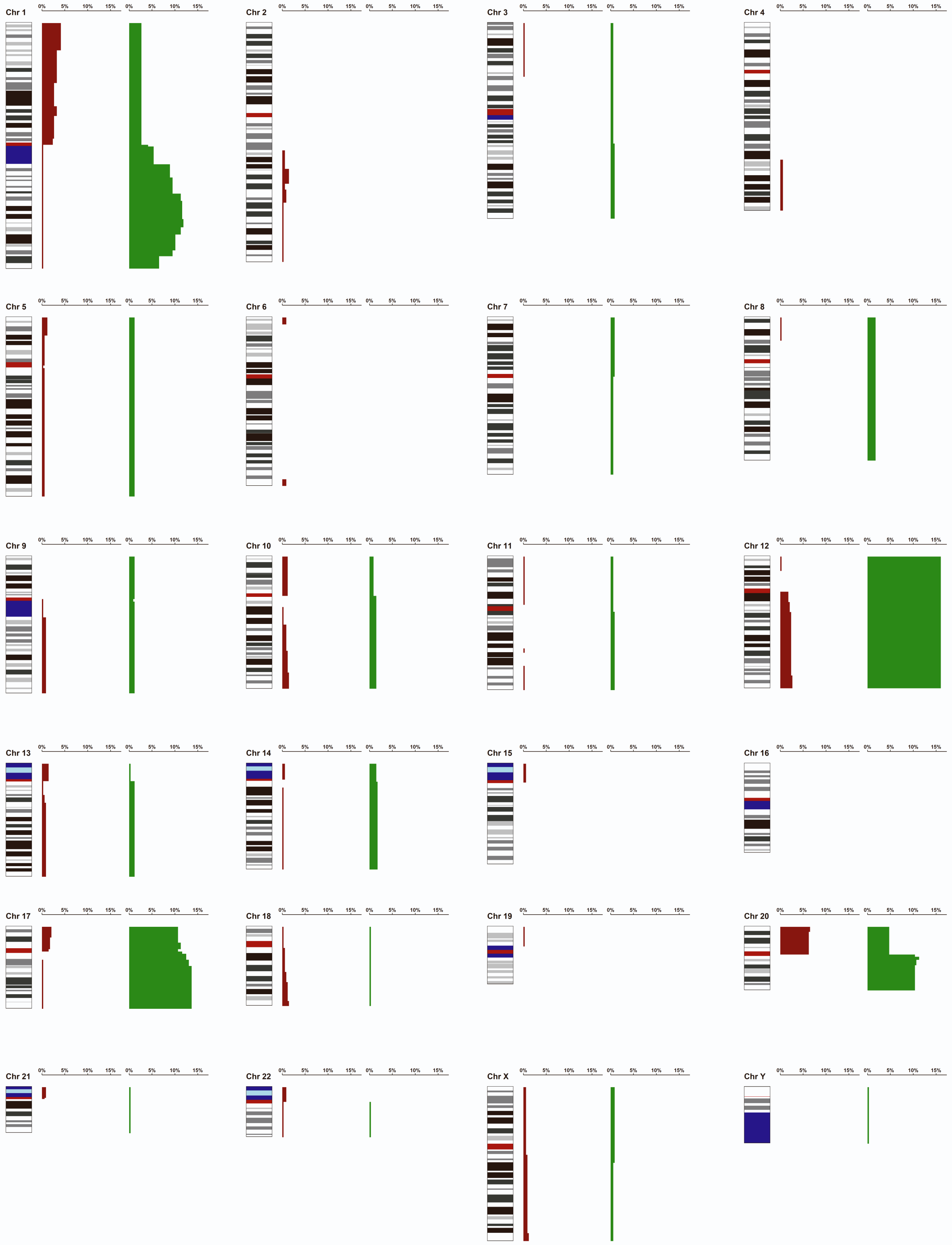

Figure S3

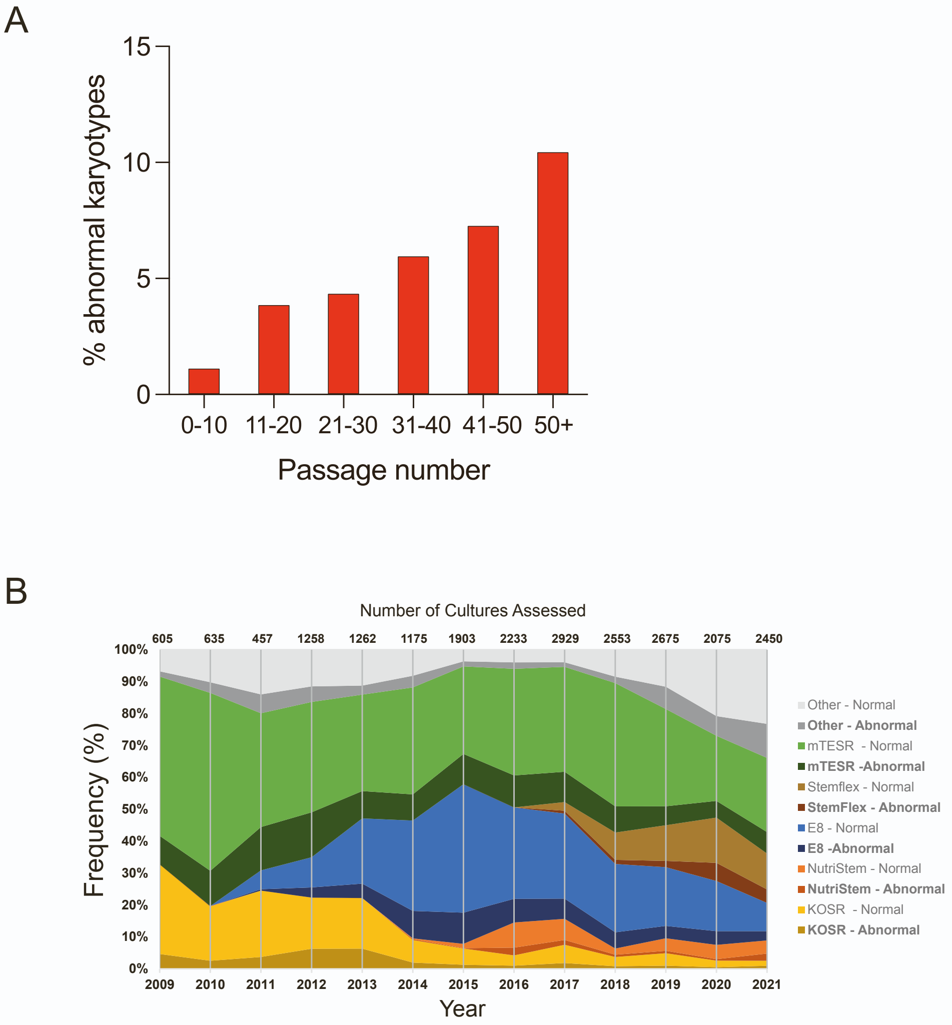

A
% abnormal karyotypes
15
10
5
0
0-10
11-20
21-30
31-40
41-50
50+
Passage number
B
Number of Cultures Assessed
605
635
457
1258
1262
1175
1903
2233
2929
2553
2675
2075
2450
Frequency (%)
100%
90%
80%
70%
60%
50%
40%
30%
20%
10%
0%
2009
2010
2011
2012
2013
2014
2015
2016
2017
2018
2019
2020
2021
Year
Other - Normal
Other - Abnormal
mTESR - Normal
mTESR -Abnormal
Stemflex - Normal
StemFlex - Abnormal
E8 - Normal
E8 -Abnormal
NutriStem - Normal
NutriStem - Abnormal
KOSR - Normal
KOSR - Abnormal

Figure S4

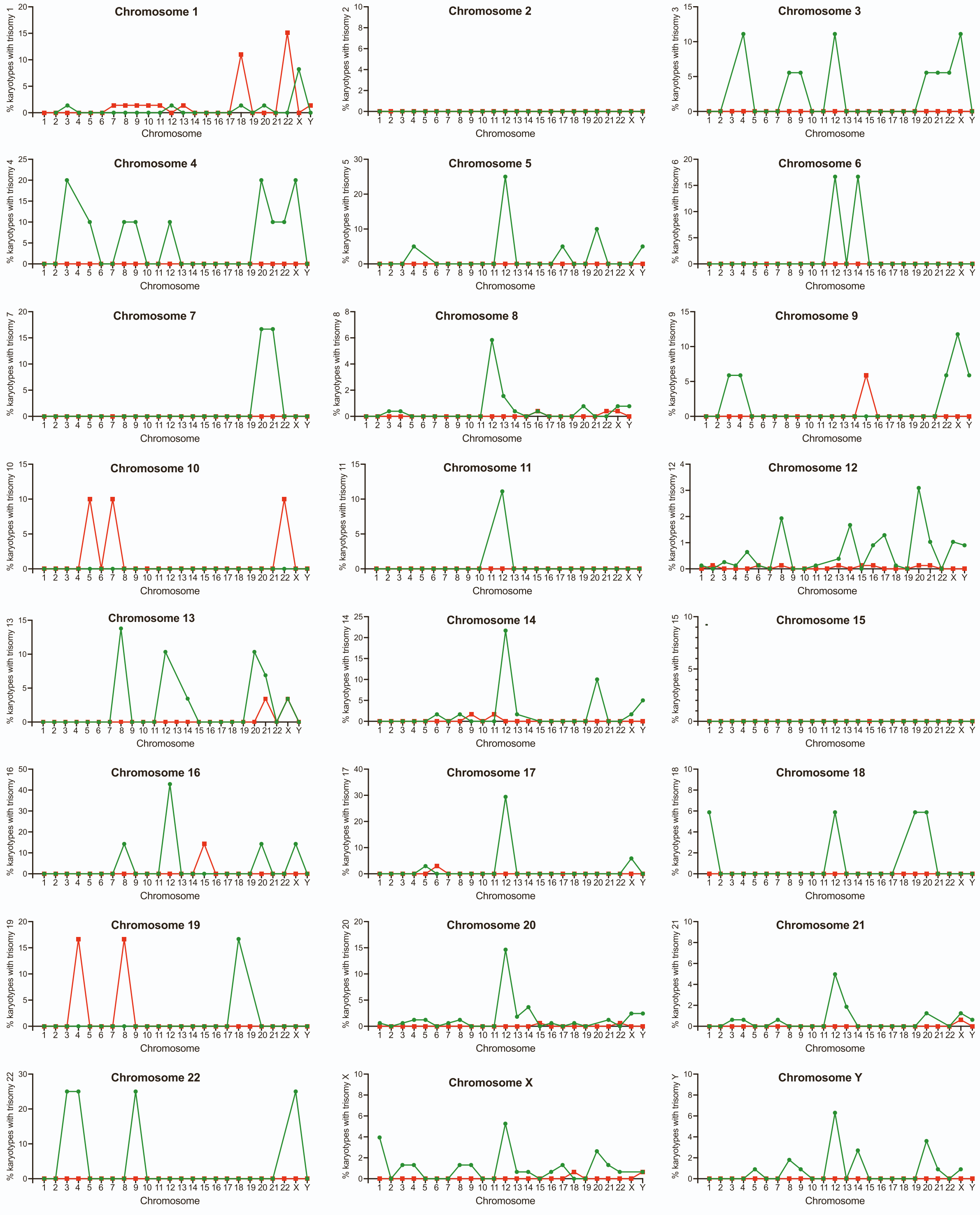

Chromosome 1
Chromosome 2
Chromosome 3
Chromosome 4
Chromosome 5
Chromosome 6
Chromosome 7
Chromosome 8
Chromosome 9
Chromosome 10
Chromosome 11
Chromosome 12
Chromosome 13
Chromosome 14
Chromosome 15
Chromosome 16
Chromosome 17
Chromosome 18
Chromosome 19
Chromosome 20
Chromosome 21
Chromosome 22
Chromosome X
Chromosome Y
% karyotypes with trisomy 1
% karyotypes with trisomy 2
% karyotypes with trisomy 3
% karyotypes with trisomy 4
% karyotypes with trisomy 5
% karyotypes with trisomy 6
% karyotypes with trisomy 7
% karyotypes with trisomy 8
% karyotypes with trisomy 9
% karyotypes with trisomy 10
% karyotypes with trisomy 11
% karyotypes with trisomy 12
% karyotypes with trisomy 13
% karyotypes with trisomy 14
% karyotypes with trisomy 15
% karyotypes with trisomy 16
% karyotypes with trisomy 17
% karyotypes with trisomy 18
% karyotypes with trisomy 19
% karyotypes with trisomy 20
% karyotypes with trisomy 21
% karyotypes with trisomy 22
% karyotypes with trisomy X
% karyotypes with trisomy Y
Chromosome
1 2 3 4 5 6 7 8 9 10 11 12 13 14 15 16 17 18 19 20 21 22 X Y

Figure S5

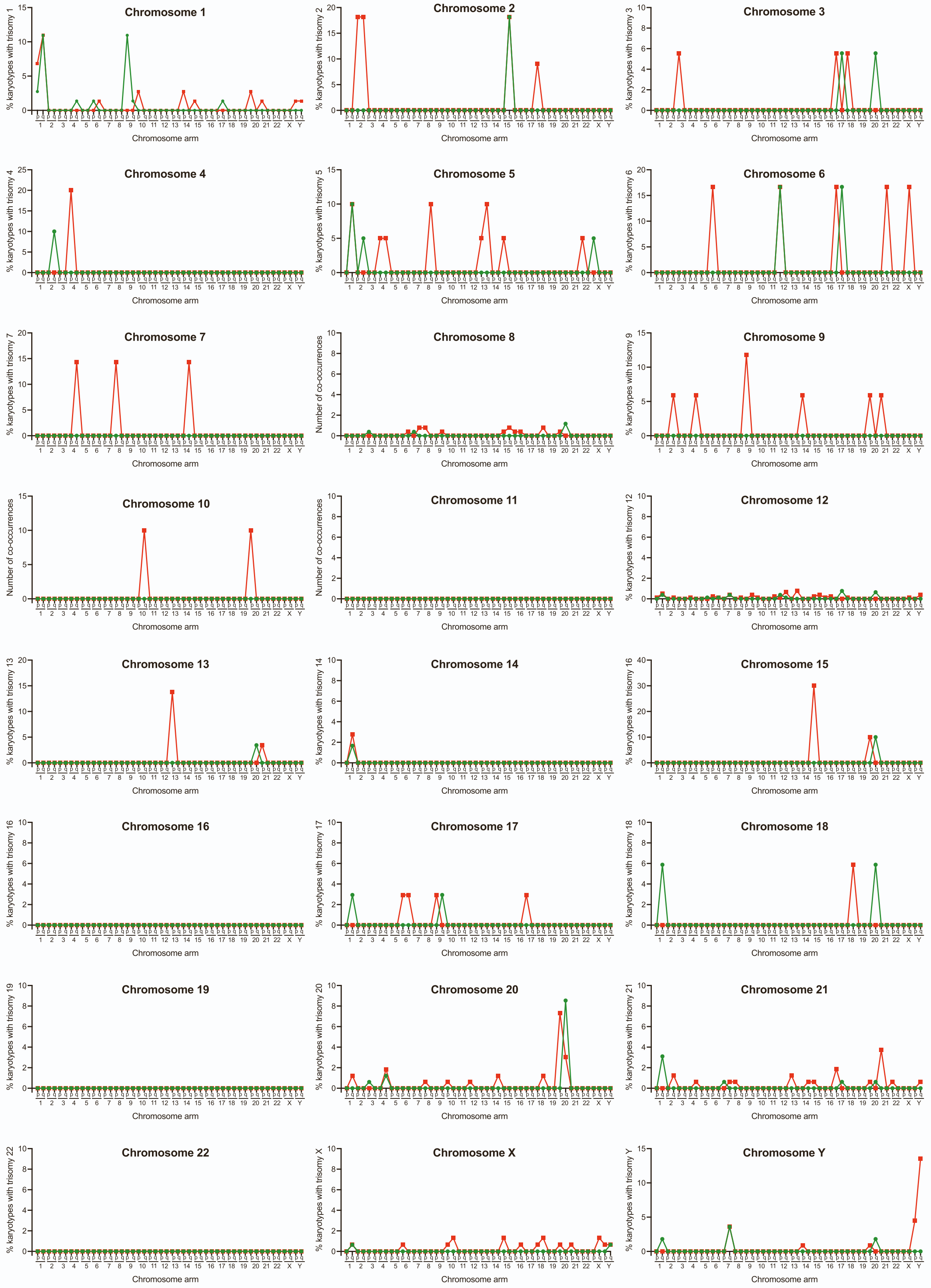

Chromosome 1
% karyotypes with trisomy 1
Chromosome 2
% karyotypes with trisomy 2
Chromosome 3
% karyotypes with trisomy 3
Chromosome 4
% karyotypes with trisomy 4
Chromosome 5
% karyotypes with trisomy 5
Chromosome 6
% karyotypes with trisomy 6
Chromosome 7
% karyotypes with trisomy 7
Chromosome 8
Number of co-occurrences
Chromosome 9
% karyotypes with trisomy 9
Chromosome 10
Number of co-occurrences
Chromosome 11
Number of co-occurrences
Chromosome 12
% karyotypes with trisomy 12
Chromosome 13
% karyotypes with trisomy 13
Chromosome 14
% karyotypes with trisomy 14
Chromosome 15
% karyotypes with trisomy 16
Chromosome 16
% karyotypes with trisomy 16
Chromosome 17
% karyotypes with trisomy 17
Chromosome 18
% karyotypes with trisomy 18
Chromosome 19
% karyotypes with trisomy 19
Chromosome 20
% karyotypes with trisomy 20
Chromosome 21
% karyotypes with trisomy 21
Chromosome 22
% karyotypes with trisomy 22
Chromosome X
% karyotypes with trisomy X
Chromosome Y
% karyotypes with trisomy Y
Chromosome arm

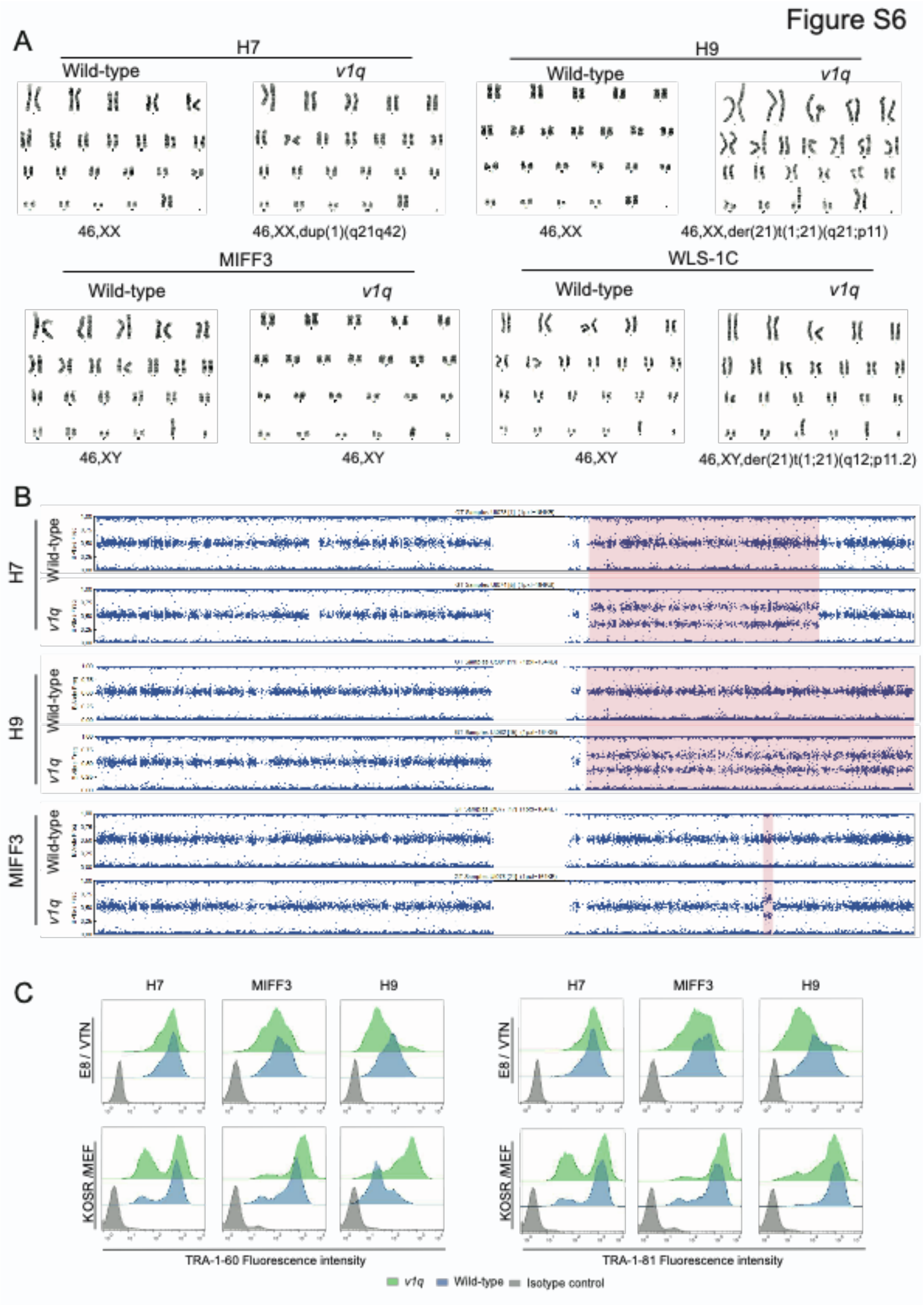
Figure S6
A
H7
Wild-type
v1q
46,XX
46,XX,dup(1)(q21q42)
H9
Wild-type
v1q
46,XX
46,XX,der(21)t(1;21)(q21;p11)
MIFF3
Wild-type
v1q
46,XY
46,XY
WLS-1C
Wild-type
v1q
46,XY
46,XY,der(21)t(1;21)(q12;p11.2)
B
H7
Wild-type
v1q
H9
Wild-type
v1q
MIFF3
Wild-type
v1q
C
H7
MIFF3
H9
E8 / VTN
KOSR MEF
TRA-1-60 Fluorescence intensity
TRA-1-81 Fluorescence intensity
v1q
Wild-type
Isotype control

Figure S7

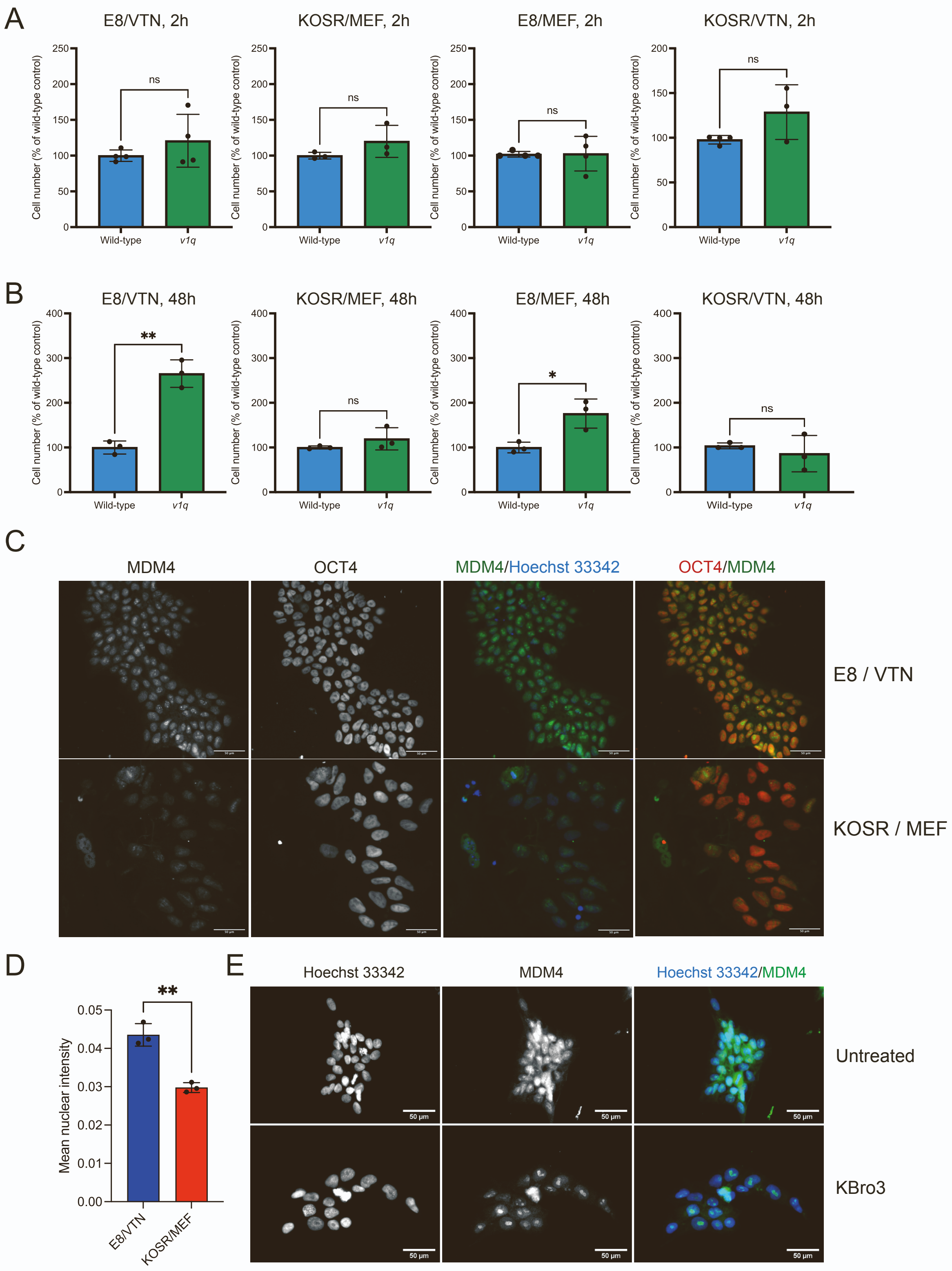

A
E8/VTN, 2h
KOSR/MEF, 2h
E8/MEF, 2h
KOSR/VTN, 2h
Cell number (% of wild-type control)
ns
Wild-type
v1q
B
E8/VTN, 48h
KOSR/MEF, 48h
E8/MEF, 48h
KOSR/VTN, 48h
**
*
C
MDM4
OCT4
MDM4/Hoechst 33342
OCT4/MDM4
E8 / VTN
KOSR / MEF
D
Mean nuclear intensity
E8/VTN
KOSR/MEF
E
Hoechst 33342
MDM4
Hoechst 33342/MDM4
Untreated
KBro3
50 µm

SUPPLEMENTAL FIGURE LEGENDS

Figure S1 (related to Figure 1 and Table S2). An ideogram depicting the frequency at which a particular cytoband is represented in abnormal hPSC karyotypes in the WiCell dataset. Green bars next to a chromosome represent a gain of the corresponding chromosomal region. Red bars represent losses.

Figure S2 (related to Figure 1 and Table S2). An ideogram depicting the frequency at which a particular cytoband is represented in abnormal hPSC karyotypes in the CSCB dataset. Green bars next to a chromosome represent a gain of the corresponding chromosomal region. Red bars represent losses.

Figure S3 (related to Figures 1 and 3, and Table S1).

A. Effects of passaging numbers and cell line identity on the appearance of aberrations. Analysis of the appearance of aberrations in relation to passage numbers shows a trend in the increase of abnormal karyotypes with an increase in passage numbers.

B. The relative frequency of the most used media in the WiCell dataset over time, shaded by normal and abnormal cultures identified.

Figure S4 (related to Figure 1 and Table S1). Co-occurrences of a trisomy of a specific chromosome with another chromosome trisomy.

Figure S5 (related to Figure 1 and Table S1). Co-occurrences of a trisomy of a specific chromosome with a partial gain or loss of a chromosome.

Figure S6 (related to Figure 4). Genetic and phenotypic analysis of the lines used in the study.

A. Examples of karyotypes of H7, H9, MIFF3 and WLS-1C wild-type and *v1q* sublines.

B. SNP array analysis of H7, H9, MIFF3 wild-type and *v1q* sublines confirmed the presence of chromosome 1q gain. MIFF3 has a relatively small amplification of chromosome 1q that is not readily detectable by G-banding.

C. Wild-type and *v1q* cells display overall similar levels of pluripotency-associated markers TRA-1-60 and TRA-1-81 in E8/VTN and in KOSR/MEF conditions.

Figure S7 (related to Figures 6 and 7). KOSR-based medium, rather than MEFs, causes diminished advantage of *v1q* cells in KOSR/MEF compared to E8/VTN.

A. Similar numbers of H9 wild-type and *v1q* hPSCs attach regardless of the medium (E8 and KOSR) or the matrix (VTN and MEFs) at 2h post-plating. Data shown are the mean ± SD of three independent experiments. ns, non-significant; Unpaired *t* test.

B. Numbers of H9 *v1q* hPSCs are higher than H9 wild-type cells in E8/VTN and E8/MEF conditions but are similar to wild-type cells in KOSR/MEF and E8/MEF condition at 48h post-plating. Data shown are the mean ± SD of three independent experiments. ns, non-significant, *p<0.05, ***p<0.001; Unpaired *t* test.

C. MDM4 localisation is more nuclear in hPSCs grown in E8/VTN compared to KOSR/MEF, related to the induction of genome damage. Representative images of H9 hPSCs grown in E8/VTN (upper panels) or KOSR/MEF (lower panels) stained with antibodies against MDM4 and OCT4 (POU5F1). The nuclei are counterstained with Hoechst 33342. Scale bar: 50μm.

D. Quantification of the MDM4 nuclear expression in E8/VTN versus KOSR/MEF condition in H9 line. Data shown are the mean ± SD of three independent experiments. **p<0.01; Unpaired *t* test.

E. MDM4 exhibits nuclear localisation upon the induction of genome damage with potassium bromate (KBrO3). Representative images of hPSCs grown in E8/VTN (untreated) and in E8/VTN supplemented with 500nM KBrO3, stained for MDM4. Nuclei are counterstained with Hoechst 33342. Scale bar: 50μm.

SUPPLEMENTAL EXPERIMENTAL PROCEDURES

Karyotyping database assembly and analyses

Karyotyping data analysed in this study was collected by WiCell (Madison, USA) and the Centre for Stem Cell Biology (CSCB) (Sheffield, UK) over a period of 2009-2021 and 2002-2019, respectively. While CSCB data was mainly generated from hPSC lines grown in-house (around 80 different lines), WiCell data contained hPSC samples that were grown not only in-house but also submitted to WiCell for cytogenetic analysis by different laboratories (estimated to contain over 1500 different cell lines). The WiCell dataset builds on the dataset reported in (Taapken et al., 2011) (~1,700 karyotypes) and adds a further 10 years of data collection (~20,500 karyotypes), which equates to more than a 10-fold increase in cytogenetic data analysed.

The data was stored in a format of standard International System for Human Cytogenetic Nomenclature (ISCN), with some associated data, such as the data of submission, to the sample. WiCell data also contained media/matrix information according to customer submission. This information was screened, and blanket terms were assigned to both media and matrix for the purposes of tracking changes related to these variables but also to respect the anonymity of the WiCell customers. The CSCB dataset was curated to remove repeated sampling of the same cell line, especially given the CSCB's active work using genetically variant hPSC lines. This same curation was not possible for the WiCell datasets due to the anonymised data. Errors in the karyotype nomenclature were corrected where possible and speculative changes denoted with question marks (?) were treated as correct calls. The karyotypes for mosaic cultures were split up for classification of karyotypic changes but kept together when assessing the number of cultures with abnormal karyotypes. In assessing the matrices for growing the cells Cultrex, which had only a ~12% abnormality rate, was omitted from the matrix comparison as there was evident sampling bias with ~94% of karyotypes of cells grown on Cultrex coming from just one institute.

Once curated, the data was run through CytoGPS(Abrams et al., 2019) (http://cytogps.org/) or through their offline package (https://github.com/i2-wustl/CytoGPS) which can read written karyotypes and translate them into information of losses and gains for chromosomal regions. The new dataset was put into the R package, RCytoGPS(Abrams et al., 2021), for further analysis and visualisation using ggplot2 (https://ggplot2.tidyverse.org/) and magick (https://cran.r-project.org/web/packages/magick/index.html).

To facilitate further exploration of our collated dataset, we also compiled a browser-based database termed KaryoBrowser (https://karyo.group.shef.ac.uk/). The browser-based database was assembled using RMarkdown (https://bookdown.org/yihui/rmarkdown/) and FlexDashboard (https://pkgs.rstudio.com/flexdashboard/). CytoGPS data was used for plotting interactive ideograms using ChromoMap (Anand and Rodriguez Lopez, 2022) and bar charts using rplotly (https://plotly-r.com). A Cascading Style Sheets (CSS) structure was added to allow for browser-based selection of parameters. Using KaryoBrowser, the collated datasets in this study can be inspected and filtered based on different parameters, including culture media, matrix, whether the lines are embryonic or induced PSCs and their sex. Interactive ideograms can be generated as well as bar charts detailing the chromosomal alterations associated with each parameter. The filtered data containing the karyotypes can also be exported for further analysis.

Human pluripotent stem cell (hPSC) lines

Wild-type hPSCs used in this study were H7 (WA07) (Thomson et al., 1998) (RRID:CVCL_9772), H9 (WA09) (Thomson *et al.*, 1998) (CVCL_9773), MIFF-3 (Desmarais et al., 2016) (CVCL_1E70) and WLS-1C (Chang et al., 2013). Wild-type sublines were karyotypically normal (based on at least 20 metaphases analysed by G-banding of cell banks prior to experiments and did not possess a commonly gained 20q11.21 copy number variant (as determined by quantitative PCR for copy number changes(Baker et al., 2016; Laing et al., 2019) and/or Fluorescent In Situ Hybridisation(Baker *et al.*, 2016)).

Genetically variant hPSCs used in this study and their karyotypes were: H7 *v1q* (RRID:CVCL_A5KR) ([46,XX,dup(1)(q21q42)] (20 metaphases analysed), H9 *v1q* [46,XX,der(21)t(1;21)(q21;p11)] (20 metaphases analysed), MIFF-3 *v1q* [46,XY] (20 metaphases analysed) and WLS-1C *v1q* [46,XY,der(21)t(1;21)(q12;p11.2) (20 metaphases analysed). Of note, although MIFF-3 *v1q* appeared diploid by G-banding analysis, a duplication of q32.1 on chromosome 1 was detected by qPCR and

confirmed by SNParray analysis. The H7 *v1q* cell line and the fluorescently labelled H7 subline, H7-RFP, were established and described previously (Price et al., 2021). The WLS-1C *v1q* cells were kindly provided by STEMCELL Technologies. H9 *v1q* and MIFF-3 *v1q* were established in this study by cloning out spontaneously arising variants from mosaic cultures using single cell deposition by fluorescent activated cell sorting. Single cells from mosaic cultures were sorted directly into individual wells of a 96 well plate using a BD FACS Jazz (BD Biosciences) and cultured to form colonies over 2-3 weeks. The resulting colonies were expanded in culture and subsequently frozen to establish cell banks. At the time of freezing, sister flasks were sent for either karyotyping by G-banding and/or SNP array profiling and assessment of the relative copy number of commonly identified genetic changes by qPCR (Baker *et al.*, 2016; Laing *et al.*, 2019). To facilitate cell competition assays, fluorescently labelled sublines of wild-type H9 and MIFF-3 and *v1q* lines were generated, as previously described (Price *et al.*, 2021). Briefly, cells were transfected with pCAG-H2B-RFP plasmid (a kind gift from Dr Jie Na, Tsinghua University, Beijing) or pCAG-H2B-GFP plasmid (Price and Barbaric, 2022) (Addgene, cat. no. 184777) using Neon Transfection System (Cat. # MPK10025; Thermo Fisher Scientific). The stably transfected cells were selected by growing in medium supplemented with puromycin (Cat. # A11138; Thermo Fisher Scientific), bulk-sorted to enrich for RFP or GFP- expressing cells and subjected to karyotyping by G-banding and/or SNParray and qPCR analysis for the presence of 1q gain.

Banks of wild-type cells were made at 17 passages from derivation from H7, 14 passages from H9, 29 passages from MIFF-3 and 38 passages for WLS-1C. Banks of H7 and H9 fluorescently labelled cells were made at 24 and 47 passages, respectively. For variant cells, banks were made at 32 passages for H7 *v1q*, 46 passages for H9 *v1q* and 56 passages for WLS-1C *v1q*. Upon defrosting from a bank, cells were used for experiments after 3 passages back in culture up to passage 10. After this a new vial would be defrosted from the master banks. The majority of experiments took place between passages 4 and 7 upon defrosting the bank.

Human pluripotent stem cell culture

For E8/VTN culture conditions, hPSC were grown on vitronectin (VTN-N) (Cat. # A14700, Life Technologies)-coated flasks (5 µg/ml in Dulbecco's phosphate buffered saline (PBS) without Calcium and Magnesium) in modified E8 medium (Chen et al., 2011) prepared in house, consisting of DMEM/F12 (Cat. # D6421; Sigma-Aldrich) supplemented with 14 µg/l sodium selenium (Cat. # S5261; Sigma-Aldrich), 19.4 mg/l insulin (Cat. # A11382IJ; Thermo Fisher Scientific), 1383 mg/l $NaHCO_3$ (Cat. # S5761; Sigma-Aldrich), 10.7 mg/l transferrin (Cat. # T0665; Sigma-Aldrich), 10 ml/l Glutamax (Cat. # 35050038; Thermo Fisher Scientific), 40µg/l FGF2-3 (Cat. # Qk053; Qkine) and 2 µg/l TGFβ1 (Cat. # 100-21; Peprotech). For time lapse experiments, E8 was prepared using DMEM/F12 without phenol red (Cat. # D6434; Sigma-Aldrich).

For KOSR/MEF culture conditions, hPSCs were grown on a layer of mitotically inactivated mouse embryonic fibroblasts (feeders) in KOSR-based medium consisting of KnockOut DMEM (Cat. # 10829018; Thermo Fisher Scientific), 20% KnockOut Serum Replacement (Cat. # 10828028 Thermo Fisher Scientific), 1x Non-essential amino acids (Cat. # 12084947; Fisher Scientific), 1mM L-Glutamine (Cat. # 25030081; Thermo Fisher Scientific), 0.1mM 2-mercaptoethanol (Cat. # 11528926; Fisher Scientific) and 8ng/ml FGF2-G3 (Cat. # Qk053; QKine).

Cells were fed daily and maintained at 37°C under a humidified atmosphere of 5% CO_2 in air. Routine passaging was performed every 4-5 days using ReLeSR (Cat. # 05873; STEMCELL Technologies) according to manufacturer's instructions in E8/VTN (1:6 ratio for wild-type cells and 1:12 for variant 1q) or by treatment with Collagenase IV (Cat. # MB-121-0100; Cambridge Bioscience Ltd) and manually scraping of colonies grown in KOSR/MEF. No antibiotics were used during the culture. Cells in culture were checked for mycoplasma quarterly, using the Mycoplasma Detection Kit (Cat. # rep-mysnc-100; InvivoGen).

For freezing, cultures were split in a 1:4 split ratio in STEM-cellbanker (Cat. # 11924; AMSBIO). Lines were defrosted in mTeSR1 (STEMCELL Technologies) with 10uM Y-27632. After one day Y-27632 was removed and after two days the medium was replaced with E8.

Karyotyping by G-banding

Karyotyping by G-banding for lines used in experiments in this study was performed by the Sheffield Diagnostic Genetics Service (https://www.sheffieldchildrens.nhs.uk/sdgs/), as previously described

(Baker and Barbaric, 2022; Baker *et al.*, 2016). Briefly, hPSCs were treated with 0.1 µg/ml KaryoMAX Colcemid (Cat. # 15212012; Thermo Fisher Scientific) for up to 4h. The cells were then harvested with trypsin, re-suspended in pre-warmed 0.0375M KCl hypotonic solution. After incubating for 10 min at room temperature, cells were pelleted and resuspended in fixative (3:1 methanol:acetic acid). Metaphase spreads were prepared on glass microscope slides and G-banded by brief exposure to trypsin and stained with 4:1 Gurr's/Leishmann's stain (Sigma-Aldrich). Slides were scanned, metaphase images captured and analysed using the Leica Biosystems Cytovision Image Analysis system (version 7.3.2 build 35).

SNP arrays

SNP array profiling was performed on Infinium Global Screening Array-24 v3.0 BeadChip GSA. Array data was obtained from the HuGe-F as a Genome Studio vs. 2.0.4 (Illumina, Eindhoven, The Netherlands) project using the hg38 reference genome, as described previously(Timmerman et al., 2021).

Competition assays

To assess selective advantage of variants and MDM4 overexpressing cells, mixing experiments were performed using matched fluorescently labelled and unlabelled lines, as follows: H7 *v1q* with H7-RFP cells, H9 *v1q* with H9-RFP cells, MIFF3 *v1q*-H2B-GFP with MIFF3 counterparts, and MIFF3-GFP cells with MIFF3 *v1q* or MIFF3-MDM4 cells. Cells were harvested to single cells by treating with Accutase (A6964; Sigma-Aldrich) and counted. Cells were then mixed to contain 10% *v1q* cells, or MDM4 overexpressing cells, with the respective wild-type population. Cell mixes were plated into either E8/VTN or KOSR/MEF conditions with the addition of 10µM Y-27632 for 24h. Cultures were grown and passaged as described above. At each passage, one flask was kept for further passaging while a parallel flask was harvested for assessment of the ratio of individual sublines using flow cytometry.

Clonogenic assays

Cells were harvested by treating with Accutase (A6964; Sigma-Aldrich) at 37°C for 10 min to create a single cell suspension. Cells were washed with DMEM/F12, pelleted at 200*g* and resuspended in fresh DMEM/F12. Cells were then seeded at a density of ~500 cells per cm^2 in 24 well plates, which had been pre-coated with either vitronectin or plated with mouse embryonic fibroblasts (MEF), and incubated with 0.5ml of pre-warmed media containing 10µM Y-27632 (Cat. # A11001-10; Generon). After two days, a fresh 0.5ml of media containing 10µM Y-27632 was added on top of the existing media. After four days, the media was fully replaced with 0.5ml of media without Y-27632. On day 5, cells were fixed using 4% PFA and stained for NANOG (Cat. # 4903; Cell Signalling Technology) and nuclei were counterstained with Hoechst 33342. Images were taken on the InCell 2200 and tiled across the well. The tiled images were stitched together for analysis and colonies were counted from images using a CellProfiler (Carpenter et al., 2006) pipeline.

Growth curve analysis

Initial assessment of population growth of wild-type and *v1q* cells was done using MIFF3 H2B-GFP and MIFF3 *v1q* H2B-GFP cells plated at 30,000 cells per cm^2 in 96 well plates. Cells were plated directly into test conditions, i.e., E8/VTN or KOSR/MEF with 10µM Y-27632 (Cat. # A11001-10; Generon). After 24 hours, the media was replaced and Y-27632 was removed. Cells were imaged for GFP signal at day 0, 1, 2 and 3 using the InCell Analyzer with 16 set positions in each well. The GFP signal at each position was calculated as area of image covered and tracked over the days. The resulting average of the fields was used to calculate the fold change in growth.

To assess the effect of KOSR addition to E8, MIFF3 cells and MIFF3 *v1q* cells growth rate analysis was performed, as previously described[7]. In brief, cells were plated at 30,000 cells per cm^2 in 96 well plates. Cells were plated directly into test conditions, i.e., E8/VTN, KOSR/MEF or E8+20% KOSR/VTN with 10µM Y-27632 (Cat. # A11001-10; Generon). After 24 hours, Y-27632 was removed and the media replenished daily. Plates were fixed daily with 4% PFA and nuclei counterstained with Hoechst 33342

prior to imaging on InCell Analyzer 2200. The resulting images were analysed using a CellProfiler pipeline (Carpenter *et al.*, 2006) to calculate cell numbers.

Time-lapse analysis

Time-lapse microscopy was performed at 37°C and 5% CO_2 using a Nikon Biostation CT. To perform lineage analysis, cells were imaged every 10 min for 96 hours using 10x air objective. Image stacks were compiled in CL Quant (Nikon) and exported to FIJI (Image J) (Schindelin et al., 2012) for analysis. Lineage trees were constructed manually from FIJI movies. Individual cells were identified in the first frame and then tracked in each subsequent frame until their death, division or the end of the 72 hours. The timing of cell death or division for each cell was noted and then used to reconstruct lineage trees of founder cells using Interactive Tree Of Life (iTOL) (Letunic and Bork, 2007) software. Tracking cells beyond 72 hours was not feasible as variant cultures were becoming too dense. Lineage trees were used to calculate the cell cycle time and the proportion of daughter cells surviving/dying following the division, as previously described (Barbaric et al., 2014).

Flow cytometry

To assess the ratio of individual sublines in mixing experiments, cells were harvested with TrypLE (Cat. # 11528856; Thermo Fisher Scientific) or Accutase (A6964; Sigma-Aldrich) and resuspended in DMEM/F12. The sample was then pelleted by centrifugation at 200 *g* for 5 min and subsequently fixed with 4% PFA for 10 min. The sample was washed with PBS and stored at 4°C until analysis. Samples were run on the BD FACS Jazz (BD Biosciences) flow cytometer to assess ratios of unlabelled to labelled. Gates were set based on 100% labelled and 100% unlabelled samples.

For analysis of pluripotency-associated surface antigens, cells were harvested with TrypLE (Cat. # 11528856; Thermo Fisher Scientific) and resuspended in PBS supplemented with 10% Foetal Calf Serum (FCS) at $1x10^7$ cells/mL. Primary antibodies SSEA-3(Shevinsky et al., 1982), TRA-1-81(Andrews et al., 1984) and TRA-1-60 (Andrews *et al.*, 1984), prepared in-house as described previously (Draper et al., 2002; International Stem Cell et al., 2007), were added to cells suspension and incubated for 30 min at 4°C. After washing with PBS supplemented with 10% FCS, cells were incubated with secondary antibody (Goat anti-Mouse AffiniPure IgG+IgM (H+L), Cat. # 115-605-044-JIR; Stratech) at 1:200 for 30 min at 4°C in the dark. After washing twice with PBS supplemented with 10% FCS, analysed on BD FACS Jazz (BD Biosciences). Baseline fluorescence was set using the isotype control antibody P3X, an antibody secreted from the parental myeloma cell line P3X6Ag8(Kohler and Milstein, 1975).

Flow cytometry for cleaved caspase-3 was performed to assess levels of apoptotic cells in cultures, as previously described (Price and Barbaric, 2022; Price *et al.*, 2021). In brief, the old media, containing apoptotic cells which had detached from the flask, was collected into a 15ml Falcon tube. The remaining cells within the flask were harvested with TrypLE (Cat. # 11528856; Thermo Fisher Scientific) and added to the 15ml tube containing the collected culture medium and apoptotic cells. The collated sample was pelleted by centrifugation at 270 *g* for 5 min and subsequently fixed with 4% PFA. Cells were permeabilised with 0.5% Triton X-100 in PBS and then incubated with anti-cleaved caspase-3 primary antibody (Cat. # 9661; Cell Signalling Technology) in blocking buffer (1% BSA and 0.3% Triton X-100 in PBS). Samples were gently agitated for 1 hour at room temperature or overnight at 4°C, prior to washing and staining with secondary antibody (Goat anti-Rabbit AffiniPure IgG+IgM (H+L), Cat. # 111-605-003-JIR; Stratech) for 1 hour at room temperature in the dark. Cells were then washed and analysed on BD FACS Jazz (BD Biosciences). Baseline fluorescence was set using secondary antibody-only stained samples.

Immunocytochemistry

Cells were fixed with 4% paraformaldehyde for 15 min at room temperature and then blocked and permeabilised with 10% foetal calf serum (FCS) supplemented with 0.2% Triton X-100 in PBS for 10 minutes RT or 1 hour at 4°C. Cells were incubated with a primary antibody in 10% FCS at room temperature for 1 hour or at 4°C overnight. After three washes with PBS, cells were incubated with a secondary antibody in 10% FCS for 1 hour. Cells were then washed with PBS and stained with Hoechst 33342. The primary antibody used were: MDM4 (Cat. #04-1556; Sigma-Aldrich), Anti-gamma H2A.X

(Phospho S139) (Cat. #ab26350; abcam), phospho-FAK (Tyr397) (Cat. #44-624G, ThermoFisher Scientific), OCT4 (Cat. #2890, Cell signalling). The secondary antibodies were: anti-mouse AF647 (Cat. # 115-605-044, Stratech), anti-mouse AF488 (Cat. # 115-545-044, Stratech), anti-mouse AF594 (Cat. #115-095-044, Stratech), anti-rabbit AF594 (Cat. # 111-585-045, Stratech), anti-rabbit AF647 (Cat. # 111-605-045, Stratech), Alexa Fluor 488 donkey anti-rabbit (Cat. #A21206, Invitrogen).

Western blotting

Cells were lysed in 1x RIPA Buffer pre-warmed to 95°C and the total protein concentration was normalised using the Pierce BCA Protein Assay (Cat. # 23227; Thermo Fisher Scientific). Proteins (20 µg, 10µg and 5 µg /sample) were resolved by SDS-PAGE on Mini-PROTEAN TGX Stain-Free Gels (Cat. # 4568086; Bio-Rad) and were run alongside a Page Ruler prestained protein ladder (Cat. # 26616; Thermo Fisher Scientific). Proteins were then transferred onto a 0.2µm PVDF membrane (Cat. # 1704156; Bio-Rad) using a Trans-Blot Turbo Transfer System (Bio-Rad). The membrane was blocked in 5% milk for one hour, washed three times with TBS-T (50 mM Tris-HCl (pH 7.5), 150 mM NaCl, 0.1% (v/v) Tween 20) and then incubated with primary antibodies for MDM4 (Cat. # 04-1556; Sigma-Aldrich) at 1:1,000 dilution, or β-ACTIN (Cat. #60008-1-Ig; Proteintech) at 1:5,000 dilution. Following three washes with TBS-T, the membrane was incubated with secondary antibody Anti-Mouse IgG (H+L), HRP conjugate Cat. # 1706516; Bio-Rad) at 1: 5,000 dilution for 1h. After three washes, immunoreactivity was visualised using Clarity Western ECL Substrate (Cat. # 1705061, Bio-Rad) and signal captured on either x-ray film or digital detection using the LI-COR C-DiGit (LI-COR Biosciences).

RNA extraction, sequencing and analysis

Three independent replicates of wild-type and *v1q* sublines of the H7 and H9 hPSC lines from E8/VTN cultures were used for RNA extraction and RNAseq analysis. Prior to RNA isolation, cells were lysed using Buffer RLT (Cat. # 79216; Qiagen) and stored at -80°C. RNA was isolated, libraries constructed and sequenced by GENEWIZ (Azenta Life Sciences, UK).

Briefly, libraries were prepared for Illumina (New England Biolabs, Ipswich, USA) and the library preparations were sequenced on an Illumina Novaseq platform (Illumina, San Diego, USA) to generate 150 bp paired-end reads. The sequencing reads were aligned to the GRCh38 human reference genome using STAR aligner v.2.5.2b. Unique gene hit counts were calculated using featureCounts from the Subread package v.1.5.2 and were normalized into transcript per million mapped reads (TPM), based on the length of the gene and reads count mapped to it. Differential gene expression analysis was performed for each pair of cell lines independently (H7-WT vs H7-*v1q*, H9-WT vs H9-*v1q*). Highly-expressed genes (expressed in the top quartile of at least half of the samples) were considered for further analyses. Gene set enrichment analysis(Subramanian et al., 2005) was performed using GSEA software 4.0.3 using the following parameters: gene set, 1000 permutations, and 'Collapse' analysis.

MDM4 siRNA knock-down

To knockdown the expression level of MDM4 in MIFF-3 *v1q* cells, we used MISSION esiRNA for MDM4 (ESIRNA HUMAN MDMX, Cat. # EHU005381-20UG; Sigma-Aldrich) and MISSION esiRNA for Renilla Luciferase as a control (ESIRNA RLUC Cat. # EHURLUC-20UG; Sigma-Aldrich). A 500µl transfection reaction included 50 nM siRNA and 5.6 µl DharmaFECT 1 Transfection Reagent (Cat. # T-2001-03; Horizon Discovery Ltd) in Opti-MEM I Reduced Serum Medium (Cat. # 10149832; Thermo Fisher Scientific). The reactions were incubated for 30 min at room temperature before mixing with 400,000 *v1q* cells in mTeSR (Cat. # 85850; STEMCELL Technologies) supplemented with 10µM Y-27632 (Cat. # A11001-10; Generon). Cells were plated into one well of 6-well plate per siRNA condition. After 18 hours the siRNA was removed, and cells were dissociated for plating into clonogenic assays. The remaining cells were pelleted and stored for qPCR analysis, as described below.

Quantitative PCR (qPCR)

Expression of MDM4 in siRNA knockdown experiments was assessed using qPCR. RNA was isolated using a Qiagen RNAeasy Plus Mini Kit (Cat. # 74134; Qiagen), and the RNA concentration and purity determined using a NanoPhotometer (Implen, Munich, Germany). cDNA was synthesised using a high-

capacity reverse transcription kit (Cat. # 4368814; Thermo Fisher Scientific). qPCR reactions were set up in triplicate, with each 10µl PCR reaction containing 1X PowerTrack SYBR Green Master Mix (Cat. # A46110; Thermo Fisher Scientific), 10µM Forward and Reverse Primers (Integrated DNA Technologies) and 10ng of cDNA. PCR reactions were run on a QuantStudio 12K Flex Thermocycler (Cat. # 4471087; Life Technologies). Following the first two steps of heating the samples to 50°C for 2 min and denaturing them at 95°C for 10 min, reactions were subjected to 40 cycles of 95°C for 15 s and 60°C for 1 min. The Ct values were obtained from the QuantStudio 12K Flex Software with auto baseline settings. Data was normalised to control GAPDH and 2^{-ddCt} calculated for relative expression in comparison to non-transfected *v1q* cells.

MDM4 overexpression

The pCAG-MDM4 expression vector was established by cloning of MDM4 sequence into a pCAG vector(Liew et al., 2007) containing a multiple cloning site (pCAG-MCS) using In-Fusion® Snap Assembly Starter Bundle kit (Cat. #638945; Takara Bio). A single restriction digest was performed on the pCAG-MCS vector using XhoI (Cat. #0146, New England Biolabs) to linearize plasmid. The MDM4 sequence in pLVX-TetOne-Puro-MDM4, kindly gifted to us by Jason Sheltzer (Cat. # 195140; Addgene), was PCR amplified and the resulting fragment cloned into the linearized pCAG-MCS vector as per manufacturer's instructions.

To generate the MIFF3 wild-type MDM4 overexpressing line, cells were transfected using the Neon Transfection System as described previously(Price and Barbaric, 2022; Price *et al.*, 2021). In brief, MIFF3 cells were dissociated to single cells using TrypLE and resuspended at $2{,}0x10^4$ cells/ml in "R buffer". Transfection was performed with 5µg of plasmid DNA using 1 pulse of 1600V, 20msec width. After electroporation, the cells were immediately transferred to a vitronectin-coated 60mm diameter culture dish (Cat. # 150288; Thermo Fisher Scientific) containing E8 media supplemented with 10µM Y-27632 (Cat. # A11001-10; Generon). To select for stably transfected cells, 48h post transfection cells were subjected to puromycin (Cat. # A11138; Thermo Fisher Scientific) drug selection. The cells were then expanded in the presence of puromycin selection and subsequently frozen to establish cell banks. At the time of freezing, cells from sister flasks were karyotyped by G-banding and assessed for the relative copy numbers of commonly identified genetic changes by qPCR (Baker *et al.*, 2016; Laing *et al.*, 2019). Upon defrosting and subsequent culture, cells were also regularly genotyped by karyotyping and screened for common genetic changes by quantitative PCR (Baker *et al.*, 2016; Laing *et al.*, 2019).

Cell attachment and survival assays in different matrix/media combinations

Cells were seeded at a density of 35,000 cells per cm^2 in 96 well plates, which had been pre-coated with either vitronectin or plated with mouse embryonic fibroblasts (MEF). Each matrix was tested in combination with E8 and KOSR-based medium. After 2 hours, some plates were fixed and stained with Phalloidin (Alexa Fluor® 647 Phalloidin, Cat. #8940, Cell Signalling Technology) and a phospho-FAK (Tyr397) antibody at 1:200 (phospho-FAK (Tyr397), Cat. #44-624G, ThermoFisher Scientific). The secondary antibody (Alexa Fluor 488 donkey anti-rabbit, Cat. #A21206, Invitrogen) was used at 1:200. Nuclei were counterstained with Hoechst 33342. The remaining plates were kept until 48 hours post-plating, then fixed and stained with Hoechst 33342. Images were taken on the InCell 2200 and analysed using Cell Profiler or ImageJ pipelines. Cells fixed at 2 hours post-plating were analysed for cell number, cell area and number of cells, whereas the cells fixed at 48 hours post-plating were analysed for cell numbers.

Genome damage induction

To induce genome damage, hPSCs plated on VTN were treated with either 10µM CPT or 500nM KBrO3 in E8 for 1 hour. Cells were then washed with media, fixed and processed for immunocytochemistry, as described above.

QUANTIFICATION AND STATISTICAL ANALYSIS

Statistical analysis of the data presented was performed using either GraphPad Prism version 9.0.2, GraphPad Software, La Jolla California USA, www.graphpad.com or Real Statistics Resource Pack for

Excel, Charles Zaiontz, www.real-statistics.com. Differences were tested by statistical tests as indicated in figure legends.

SUPPLEMENTAL REFERENCES

Abrams, Z.B., Tally, D.G., Abruzzo, L.V., and Coombes, K.R. (2021). RCytoGPS: an R package for reading and visualizing cytogenetics data. Bioinformatics *37*, 4589-4590. 10.1093/bioinformatics/btab683.

Abrams, Z.B., Zhang, L., Abruzzo, L.V., Heerema, N.A., Li, S., Dillon, T., Rodriguez, R., Coombes, K.R., and Payne, P.R.O. (2019). CytoGPS: a web-enabled karyotype analysis tool for cytogenetics. Bioinformatics *35*, 5365-5366. 10.1093/bioinformatics/btz520.

Anand, L., and Rodriguez Lopez, C.M. (2022). ChromoMap: an R package for interactive visualization of multi-omics data and annotation of chromosomes. BMC Bioinformatics *23*, 33. 10.1186/s12859-021-04556-z.

Andrews, P.W., Banting, G., Damjanov, I., Arnaud, D., and Avner, P. (1984). Three monoclonal antibodies defining distinct differentiation antigens associated with different high molecular weight polypeptides on the surface of human embryonal carcinoma cells. Hybridoma *3*, 347-361. 10.1089/hyb.1984.3.347.

Baker, D., and Barbaric, I. (2022). Characterizing the Genetic Stability of Human Naive and Primed Pluripotent Stem Cells. Methods Mol Biol *2416*, 267-284. 10.1007/978-1-0716-1908-7_17.

Baker, D., Hirst, A.J., Gokhale, P.J., Juarez, M.A., Williams, S., Wheeler, M., Bean, K., Allison, T.F., Moore, H.D., Andrews, P.W., and Barbaric, I. (2016). Detecting Genetic Mosaicism in Cultures of Human Pluripotent Stem Cells. Stem Cell Reports *7*, 998-1012. 10.1016/j.stemcr.2016.10.003.

Barbaric, I., Biga, V., Gokhale, P.J., Jones, M., Stavish, D., Glen, A., Coca, D., and Andrews, P.W. (2014). Time-lapse analysis of human embryonic stem cells reveals multiple bottlenecks restricting colony formation and their relief upon culture adaptation. Stem Cell Reports *3*, 142-155. 10.1016/j.stemcr.2014.05.006.

Carpenter, A.E., Jones, T.R., Lamprecht, M.R., Clarke, C., Kang, I.H., Friman, O., Guertin, D.A., Chang, J.H., Lindquist, R.A., Moffat, J., et al. (2006). CellProfiler: image analysis software for identifying and quantifying cell phenotypes. Genome Biol *7*, R100. 10.1186/gb-2006-7-10-r100.

Chang, W.Y., Lavoie, J.R., Kwon, S.Y., Chen, Z., Manias, J.L., Behbahani, J., Ling, V., Kandel, R.A., Stewart, D.J., and Stanford, W.L. (2013). Feeder-independent derivation of induced-pluripotent stem cells from peripheral blood endothelial progenitor cells. Stem Cell Res *10*, 195-202. 10.1016/j.scr.2012.11.006.

Chen, G., Gulbranson, D.R., Hou, Z., Bolin, J.M., Ruotti, V., Probasco, M.D., Smuga-Otto, K., Howden, S.E., Diol, N.R., Propson, N.E., et al. (2011). Chemically defined conditions for human iPSC derivation and culture. Nat Methods *8*, 424-429. 10.1038/nmeth.1593.

Desmarais, J.A., Unger, C., Damjanov, I., Meuth, M., and Andrews, P. (2016). Apoptosis and failure of checkpoint kinase 1 activation in human induced pluripotent stem cells under replication stress. Stem Cell Res Ther *7*, 17. 10.1186/s13287-016-0279-2.

Draper, J.S., Pigott, C., Thomson, J.A., and Andrews, P.W. (2002). Surface antigens of human embryonic stem cells: changes upon differentiation in culture. J Anat *200*, 249-258. 10.1046/j.1469-7580.2002.00030.x.

International Stem Cell, I., Adewumi, O., Aflatoonian, B., Ahrlund-Richter, L., Amit, M., Andrews, P.W., Beighton, G., Bello, P.A., Benvenisty, N., Berry, L.S., et al. (2007). Characterization of human embryonic stem cell lines by the International Stem Cell Initiative. Nat Biotechnol *25*, 803-816. 10.1038/nbt1318.

Kohler, G., and Milstein, C. (1975). Continuous cultures of fused cells secreting antibody of predefined specificity. Nature *256*, 495-497. 10.1038/256495a0.

Laing, O., Halliwell, J., and Barbaric, I. (2019). Rapid PCR Assay for Detecting Common Genetic Variants Arising in Human Pluripotent Stem Cell Cultures. Curr Protoc Stem Cell Biol *49*, e83. 10.1002/cpsc.83.

Letunic, I., and Bork, P. (2007). Interactive Tree Of Life (iTOL): an online tool for phylogenetic tree display and annotation. Bioinformatics *23*, 127-128. 10.1093/bioinformatics/btl529.

Liew, C.G., Draper, J.S., Walsh, J., Moore, H., and Andrews, P.W. (2007). Transient and stable transgene expression in human embryonic stem cells. Stem Cells *25*, 1521-1528. 10.1634/stemcells.2006-0634.

Price, C.J., and Barbaric, I. (2022). Assessing Cell Competition in Human Pluripotent Stem Cell (hPSC) Cultures. Curr Protoc *2*, e435. 10.1002/cpz1.435.

Price, C.J., Stavish, D., Gokhale, P.J., Stevenson, B.A., Sargeant, S., Lacey, J., Rodriguez, T.A., and Barbaric, I. (2021). Genetically variant human pluripotent stem cells selectively eliminate wild-type counterparts through YAP-mediated cell competition. Dev Cell *56*, 2455-2470 e2410. 10.1016/j.devcel.2021.07.019.

Schindelin, J., Arganda-Carreras, I., Frise, E., Kaynig, V., Longair, M., Pietzsch, T., Preibisch, S., Rueden, C., Saalfeld, S., Schmid, B., et al. (2012). Fiji: an open-source platform for biological-image analysis. Nat Methods *9*, 676-682. 10.1038/nmeth.2019.

Shevinsky, L.H., Knowles, B.B., Damjanov, I., and Solter, D. (1982). Monoclonal antibody to murine embryos defines a stage-specific embryonic antigen expressed on mouse embryos and human teratocarcinoma cells. Cell *30*, 697-705. 10.1016/0092-8674(82)90274-4.

Subramanian, A., Tamayo, P., Mootha, V.K., Mukherjee, S., Ebert, B.L., Gillette, M.A., Paulovich, A., Pomeroy, S.L., Golub, T.R., Lander, E.S., and Mesirov, J.P. (2005). Gene set enrichment analysis: a knowledge-based approach for interpreting genome-wide expression profiles. Proc Natl Acad Sci U S A *102*, 15545-15550. 10.1073/pnas.0506580102.

Taapken, S.M., Nisler, B.S., Newton, M.A., Sampsell-Barron, T.L., Leonhard, K.A., McIntire, E.M., and Montgomery, K.D. (2011). Karotypic abnormalities in human induced pluripotent stem cells and embryonic stem cells. Nat Biotechnol *29*, 313-314. 10.1038/nbt.1835.

Thomson, J.A., Itskovitz-Eldor, J., Shapiro, S.S., Waknitz, M.A., Swiergiel, J.J., Marshall, V.S., and Jones, J.M. (1998). Embryonic stem cell lines derived from human blastocysts. Science *282*, 1145-1147.

Timmerman, D.M., Remmers, T.L., Hillenius, S., and Looijenga, L.H.J. (2021). Mechanisms of TP53 Pathway Inactivation in Embryonic and Somatic Cells-Relevance for Understanding (Germ Cell) Tumorigenesis. Int J Mol Sci *22*. 10.3390/ijms22105377.

Supplemental Tables

1) **Table S1.** Karyotyping datasets from WiCell and CSCB. Related to Figure 1.
2) **Table S2.** CytoGPS analysis of frequencies at which cytobands are represented in abnormal hPSC karyotypes in the WiCell and CSCB dataset. Karyotypic abnormalities as percentage of abnormal and total karyotypes in the WiCell and CSCB dataset. Related to Figure 1 and Figures S1 and S2.

Video S1 (related to Figure 4). Time-lapse video of H7 (left) and H7 *v1q* cells (right) grown in E8/VTN. Images were taken every 10 min over 96h.
